# Mechanisms and Mitigation Strategies of Gas Generation in Sodium-Ion Batteries

**DOI:** 10.1007/s40820-025-01697-1

**Published:** 2025-03-10

**Authors:** Xingyan Li, Xi Chen, Meng Li, Haoran Wei, Xuming Yang, Shenghua Ye, Liewu Li, Jing Chen, Xiangzhong Ren, Xiaoping Ouyang, Jianhong Liu, Xiangtong Meng, Jieshan Qiu, Biwei Xiao, Qianling Zhang, Jiangtao Hu

**Affiliations:** 1 Graphene Composite Research Center, College of Chemistry and Environmental Engineering, Shenzhen University, Shenzhen, 518060 People’s Republic of China; 2 GRINM (Guangdong) Research Institute for Advanced Materials and Technology, Foshan, 528051 Guangdong People’s Republic of China; 3 School of Materials Science and Engineering, Xiangtan University, Xiangtan, 411105 People’s Republic of China; 4 College of Chemical Engineering, State Key Laboratory of Organic-Inorganic Composites, State Key Laboratory of Chemical Resource Engineering, Beijing University of Chemical Technology, Beijing, 100029 People’s Republic of China

**Keywords:** Sodium-ion batteries, Gas evolution mechanism, Inhibition strategies of gas generation, High-performance sodium-ion battery roadmap

## Abstract

The transition to renewable energy sources has elevated the importance of SIBs (SIBs) as cost-effective alternatives to lithium-ion batteries (LIBs) for large-scale energy storage. This review examines the mechanisms of gas generation in SIBs, identifying sources from cathode materials, anode materials, and electrolytes, which pose safety risks like swelling, leakage, and explosions. Gases such as CO_2_, H_2_, and O_2_ primarily arise from the instability of cathode materials, side reactions between electrode and electrolyte, and electrolyte decomposition under high temperatures or voltages. Enhanced mitigation strategies, encompassing electrolyte design, buffer layer construction, and electrode material optimization, are deliberated upon. Accordingly, subsequent research endeavors should prioritize long-term high-precision gas detection to bolster the safety and performance of SIBs, thereby fortifying their commercial viability and furnishing dependable solutions for large-scale energy storage and electric vehicles.

## Highlights

- The mechanisms and main sources of gas generation in sodium-ion batteries (SIBs) are discussed.
- In order to effectively improve the safety of SIBs, various strategies to inhibit gas generation are proposed.
- The future development direction to enhance the safety and performance of SIBs is emphasized.

## Graphical abstract

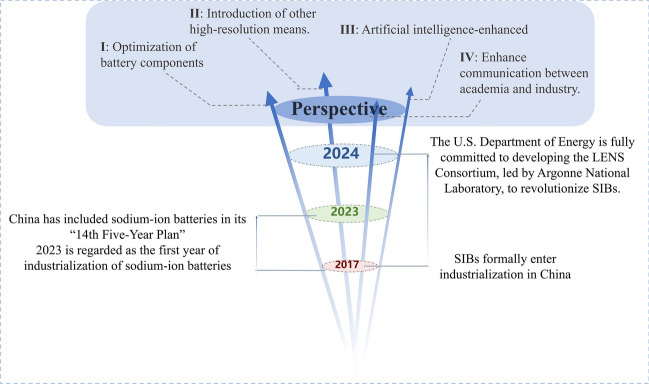

## Introduction

The transition of energy structures from traditional fossil energy to renewable energy is of immense significance for all of human society. Secondary batteries, characterized by their high energy density and long cycle life, have emerged as the most effective technology for energy storage. Among these, LIBs represent the leading electrochemical energy storage technology and have been successfully penetrated the market [1–4]. Their importance has been further emphasized by being awarded the Nobel Prize awarded in 2019 [4–9]. However, the widespread application of LIBs in large-scale energy storage systems faces challenges due to limited reserves of lithium resources and the high cost of lithium materials. In contrast, sodium possesses abundant reserves and is cost-effective with characteristics similar to lithium [10, 11]. Therefore, SIBs are considered as one of the most promising candidates to replace part of current commercial LIBs, particularly for high-power and low-temperature applications. There are some differences in the gas production behavior of sodium and lithium batteries. First of all, the gas production of sodium battery mainly comes from the instability of cathode material, the side reaction between electrode and electrolyte, and the decomposition of electrolyte under high temperature or high voltage, which produces gases including CO_2_, H_2_, and O_2_. For example, Prussian blue cathode materials in sodium batteries induce the decomposition of solvents and salts in the electrolyte under high voltage to produce gas. The gas production in lithium batteries, on the other hand, is more related to the decomposition of the electrolyte, especially under extreme conditions such as overcharging, overheating, or short-circuiting, where the solvents and additives in the electrolyte decompose to produce a large amount of combustible gases, such as CO_2_, C_2_H_4_, and C_2_H_6_. In addition, the anode electrode material (e.g., silicon anode electrode) in lithium batteries also undergoes volume changes during the charging and discharging process, resulting in the decomposition of the electrolyte to produce gas. Overall, the gas generation mechanism of sodium batteries is relatively complex, involving a variety of materials and reaction pathways, while the gas generation of lithium batteries is more focused on the decomposition of the electrolyte.

Currently, the primary focus of competition in SIB research lies in achieving higher energy density with stable cycling performance [10, 12–16]. However, the enhancement of energy density is inevitably associated with the utilization of capacity in the high-voltage region. This leads to increased reactivity of the oxygen atoms within the material matrix, as well as more intense interfacial reactions between high-valent transition metals and the electrolyte, resulting in significant gas generation issues. The accumulation of these gases leads to increased internal pressure, causing swelling, leakage, or even rupture, which deteriorates the battery’s electrochemical performance, causes physical damage, and may even lead to explosions. Therefore, improving the safety of SIBs is particularly important [17–20]. By investigating the gas generation mechanism of SIBs, we can gain a better understanding and prediction of risks while providing strong support for their development. In addition to the gas generated by the high-energy-density route mentioned above, SIBs also experience gas production during conventional testing processes, which significantly impacts the longevity and safety of SIBs [21–23].

Normally, gas generation behavior occurs during the long-term cycling [24–26], particularly under conditions of overheating and overcharging. Extensive research has been conducted on the collection and generation mechanisms of gases inside SIBs, revealing that these gases primarily originate from interfacial reactions between the electrode and electrolyte, as well as the self-decomposition of the applied electrolyte [27, 28]. Researchers have explored the gas generation mechanisms of SIBs under normal long processes and conditions of overheating and overcharging. During regular cycling, the gases generated inside the battery primarily include CO_2_, H_2_, O_2_, CO, olefins, alkanes, etc*.* [29–32]. Through isotopic labeling, titration of carbonates on the cathode surface, and electrochemical mass spectrometry, it has been demonstrated that CO_2_ and O_2_ are generated similarly to LIBs with liquid electrolytes. CO_2_ primarily originates from the decomposition of carbonates [12] on the cathode surface [12, 32, 33], while H_2_ mainly arises from trace amounts of water in the system. The generation of O_2_ is closely related to temperature and the charging state of the cathode material. Researchers attribute this phenomenon to the inadequate cyclic stability of the cathode material structure. For example, Na^+^ insertion and extraction cause structural rearrangements that result in oxygen loss in the lattice [34–37], or a transformation from a layered structure to a spinel structure. Besides, particle crack will also happen, resulting in increased contact area between the cathode and electrolyte [38–40], and consequently enhancing side reaction likelihood [41, 42]. Furthermore, compared to LIBs [43], the formation of a functional solid electrolyte interphase (SEI) in SIBs is limited by the higher solubility of SEI components (such as sodium salts). The instability of the SEI layer on the anode side leads to continuous thickening and cracking during cycling, thereby accelerating the side reactions [44]. Under conditions of overcharging or overheating, it can also lead to self-decomposition of the electrolyte system. With the accumulation of these circumstances, gases generated inside the battery gather, leading to noticeable swelling.

Importantly, many of these gases are oxidizers or flammable gases. Gas generation inside the battery has a profound impact on both electrochemical performance and safety. For instance, structural deformation of layered cathode material often coincides with the release of lattice oxygen, which can significantly impair battery capacity and charge–discharge efficiency [45]. Furthermore, the continuous accumulation of gases inside the battery may result in the reorganization or collapse of the cathode structure, rupture of the CEI film [46], and electrolyte leakage. Ultimately, this can lead to internal short-circuiting and create conditions conducive to subsequent thermal runaway, potentially resulting in combustion or explosion. Therefore, systematically summarizing the gas formation mechanisms of different battery components helps to fundamentally clarify the gas generation issues in SIBs.

To achieve high-energy-density and high-safety SIBs, this comprehensive review extensively covers the interfacial reactions between commonly utilized cathode/anode materials and electrolytes. It systematically analyzes and summarizes the associated gas generation processes, which are closely related to the degradation processes of the employed materials. Furthermore, this work compiles and discusses strategies for suppressing gas formation, thereby providing a more comprehensive understanding of gas suppression techniques. The primary objective of this review is to present a more comprehensive, efficient, and low-cost method to guide the design of SIBs with higher energy density while ensuring superior stability and safety.

## Gas Evolution from Cathode Side

Currently, layered and tunnel transition metal oxides, polyanionic cathodes, and Prussian blue analogs are the main research categories for SIB cathodes. The research progress of these materials is of great significance for improving the performance of SIB and promoting its application in the field of energy storage. However, there are still difficulties in moving from the laboratory stage to industrial scale-up production. These include complex phase changes (tremendous volume changes) of sodium-ion cathodes under high voltage, intense interface side reactions, transition metal dissolution, migration and deposition during cycling, and other issues. The macroscopic manifestations of these microscopic features in the battery are capacity fading and gas release. Obviously, the cathode material is one of the key factors in gas production. Therefore, elucidating the gas generation mechanisms of various cathode materials under different conditions is crucial to developing safer batteries.

### Layered Transition Metal Oxides

Currently, the most commonly employed cathode materials for commercial SIBs are layered transition metal oxides, which have a similar structure compared to the layered transition metal cathodes used in LIBs [12, 47, 48]. In contrast to Li^+^, Na^+^ possesses a larger ionic radius, which increases the likelihood of rearrangement or collapse of the layered structure during the intercalation and deintercalation processes of Na^+^. This structural instability can lead to lattice oxygen escape [37, 49], which reacts unfavorably with the electrolyte as oxygen molecules or radicals, thereby causing a decline in electrochemical performance, capacity reduction, and gas generation. Furthermore, during deep charge, the high-valent active transition metal ions present on the cathode surface may interact unfavorably with the electrolyte, resulting in the generation of oxygen molecules or free radicals. This interaction ultimately leads to the degradation of the electrochemical properties of the cathode and generation of gases.

The presence of residual alkali on the surface of sodium-ion layered transition metal oxides is a significant contributor to gas production in cathodes. During the sintering process, some sodium ions fail to enter the bulk phase of the cathode material, instead accumulating on the surface. This part of the Na source interacts with the environment filled with water and CO_2_ and reacts to form CO_3_^2−^ and OH^−^, forming residual Na_2_CO_3_ [50, 51] and NaOH. After a long time of cycling of the cathode material, the H_2_O and CO_2_ in the residual alkali on the surface will react with the electrolyte to generate acid as a side reaction, and react with Na_2_CO_3_ [52] and NaOH to generate H_2_O [53] and CO_2_. Introducing water and gas into battery cells can affect the performance of the battery cells and cause the battery to expand in size, causing safety issues [54, 55]. In addition, residual alkali can also hinder the transmission of sodium ions, resulting in poor surface stability of the material and rapid degradation of battery capacity. Residual bases also promote defluorination of the PVDF binder and further lead to paste gelation and particle aggregation.

The oxygen behavior in the layered transition metal oxides has been extensively studied. They observed that the primary strategy for triggering oxygen redox reactions in P2-type cathodes involves achieving a Na–O–X configuration, where X represents non-TM elements doped in the TM layer to replace TM ions (Li^+^, Na^+^, Mg^2+^, vacancies) [28, 56]. Although the oxygen redox reaction has been recognized as an effective way to overcome the capacity limitation bottleneck of both LIB and SIB cathodes, strategies aimed at enhancing oxygen redox activity inevitably led to irreversible structural changes in the cathode host. The strength of the TM–O bond or the stacking of the layers has a direct effect on the possibility of oxygen release. Strengthening the TM–O bond can improve the stability of lattice oxygen and inhibit the release of oxygen, thus improving the performance and safety of the battery. The stronger the covalency of the TM–O bond, the higher the stability of lattice oxygen, which can inhibit the over-oxidation of the lattice oxygen to O_2_.

The Na ionic radius is one aspect, but the decomposition of the electrolyte is more a result of the oxidation of high-valent transition metal ions on the surface [57–59]. High-valent metal ions (e.g., nickel, cobalt, and manganese*.*) have a high oxidation potential, resulting in the oxidative decomposition of the electrolyte in the battery charging and discharging process. Oxidation of high-valent transition metal ions leads to not only the decomposition of the electrolyte and generation of gases, but also the formation of unstable interfacial phases, thus increasing the internal resistance and self-discharge rate of the battery. In addition, the oxidation of high-valent transition metal ions can also cause decomposition of the electrolyte and the generation of gases, which leads to an increase in pressure inside the battery and affects the structural integrity and safety of the battery.

**Fig. 1:**
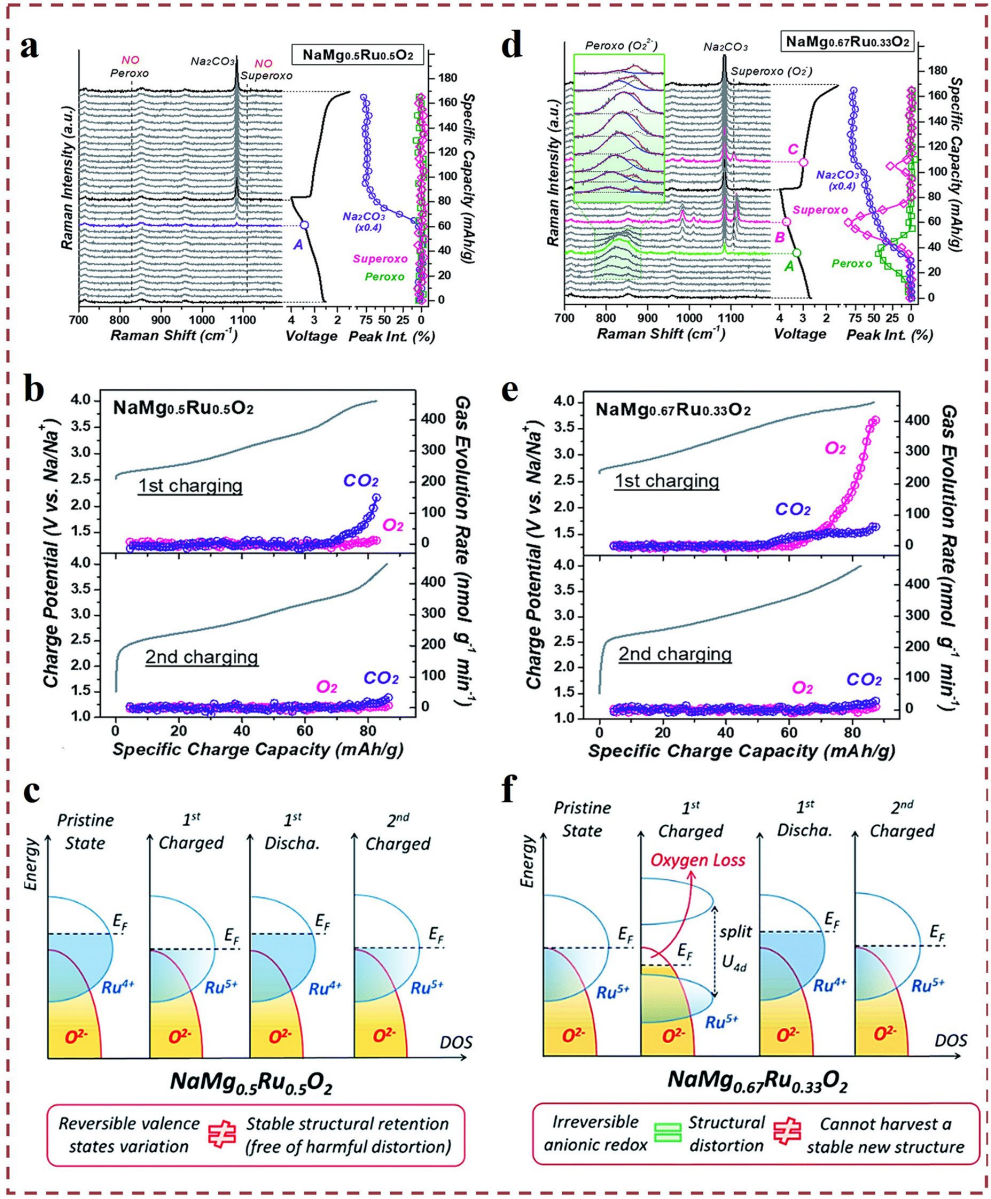
Gas production study on layered cathodes. **a** In situ Raman spectroscopy of NaMg_0.5_Ru_0.5_O_2_ during the first electrochemical cycle related to capacity. **b** Gas evolution rate results for oxygen (indicated by red circles) and carbon dioxide (indicated by purple circles) recorded by in situ DEMS during the initial and recharging of NaMg_0.5_Ru_0.5_O_2_. **c** A schematic of the density of states (DOS) plot of NaMg_0.5_Ru_0.5_O_2_ that illustrates the change in electronic structure during charging and discharging. **d** In situ Raman spectroscopy of NaMg_0.67_Ru_0.33_O_2_ during the first electrochemical cycle related to capacity. **e** Gas evolution rate results for oxygen (indicated by red circles) and carbon dioxide (indicated by purple circles) recorded by in situ differential electrochemical mass spectrometry (DEMS) during the initial and recharging of NaMg_0.67_Ru_0.33_O_2_. **f** DOS plot of NaMg_0.67_Ru_0.33_O_2_ that illustrates the change in electronic structure during charging and discharging. Reproduced with permission [21]. Copyright © 2013, Royal Society of Chemistry (Great Britain)

Li et al*.* conducted a comprehensive study on the redox reactions of layered sodium-ion cathode materials [60]. They investigated the anionic oxygen loss and its associated structural evolution in O3-type layered sodium magnesium ruthenium oxide (NaMg_0.67_Ru_0.33_O_2_) cathode materials in SIBs [21]. Using in situ Raman spectroscopy and differential electrochemical mass spectrometry (DEMS), the formation of superoxide species and oxygen were observed, indicating irreversible oxygen loss. In Fig. 1a, the capacity-dependent in situ Raman spectra was recorded from the first constant-current cycle of NaMg_0.5_Ru_0.5_O_2_, with a potential exceeding 3.5 V, and a sharp peak can be clearly seen at 1083 cm^−1^ (at point A), which was attributed to the decomposition of the propylene carbonate (PC)-based electrolyte at high polarization potentials. And the researchers found that oxygen has not been activated in the Na/ Na^+^ potential window below 4.0 V. That is, the anionic oxygen loss process does not contribute to the redox capacity in NaMg_0.5_Ru_0.5_O_2_ **(**Fig. 1c**)**. No O_2_ was detected from the DEMS (Fig. 1b), which verifies that there was no escape of lattice oxygen; however, there was CO_2_ generation at the end of charging in the high-voltage region, which the researchers attributed to the decomposition of Na_2_CO_3_ and/or electrolyte. And during the second charging process, almost no O_2_ or CO_2_ was detected, which indicates the formation of a stable cathode–electrolyte interface (CEI) protective layer after the first cycle.

During the initial charge process, the NaMg_0.67_Ru_0.33_O_2_ cathode exhibited oxygen-related anionic redox reactions. For NaMg_0.67_Ru_0.33_O_2_ (Fig. 1d–f), oxygen-related redox is activated during the initial charging process, so that oxygen-related redox activity cannot be stored in NaMg_0.67_Ru_0.33_O_2_. Therefore, at the end of the first charge, the loss and disappearance of oxygen from each oxygen-related substance are severe, resulting in complete oxygen inactivation. The process is not a reversible anionic redox, suggesting that cationic redox will be further involved. In addition, the following, X-ray photoelectron spectroscopy (XPS) observations will provide further evidence of the change in valence state during the redox process. Meanwhile, the O3 phase can be restored, indicating better structural stability, which is attributed to the structural distortion caused by initial oxygen loss. It has been clearly demonstrated in Li-rich NCM cathodes for Li-ion batteries that oxygen loss leads to chemo-mechanical breakdown of layered cathode materials [61, 62]. However, it is unfair to attribute the structural distortion (caused by lattice oxygen loss) to a deleterious structural transition throughout the layered oxide cathode. In conclusion, novel ideas are presented: Irreversible lattice oxygen loss does not necessarily correspond to deleterious structural collapse, whereas aberrations may even lead to favorable structural transformations, which instead favor structural stability in subsequent cycles.

Covalent bond strength modulation is an effective measure to inhibit irreversible oxygen release reactions at high voltages during cycling. Surface ion exchange reactions, for example, work on the principle of passivation of the cathode surface by means of surface ion exchange reactions, as in the case of the “lanthanum infiltration” treatment process, or the bimetallic strategy, which inhibits the irreversible release of lattice oxygen by enhancing the charge transfer between the transition metal and oxygen in the bulk phase and inhibiting the parasitic reaction by a protective layer of fast ionic conductors formed at the cathode interface. There are also specific applications in the field of SIBs [63–65]. Guo et al*.* prepared boron-doped sodium layered oxides to suppress the activation of lattice oxygen [34]. DEMS was employed to monitor gas evolution from the cathode materials during charge and discharge cycles in SIBs. They revealed that during the initial charge of the non-boron-doped sample, O_2_ and CO_2_ evolution were detected, indicating irreversible oxygen loss under high voltage charging, contributing to structural instability and reduced electrochemical performance. In contrast, boron-doped sample demonstrated minimal O_2_ evolution and slight CO_2_ release, suggesting effective suppression of oxygen-related side reactions through boron doping. This suppression is attributed to the formation of B–O covalent bonds in the cathode material, which enhances the oxygen ligand framework and mitigates over-oxidation during high voltage charging. Additionally, the quantitative analysis of gas evolution and estimation of X-ray absorption near edge structure (XANES) spectroscopy during initial cycling revealed the contributions to capacity from both cationic and anionic redox reactions.

### Prussian Blue Cathodes

Prussian blue and its analogs (PB and PBAs), also known as iron blue or Berlin blue, is chemically known as ferric ferrocyanide, with the chemical formula Fe_4_[Fe(CN)_6_]_3_, first discovered as blue pigments in 1706 by Johann Jacob von Diesbach and Johann Conrad Dippel [66, 67]. PBAs are widely applied across various fields, including rechargeable batteries, catalysts, biosensors, optically switchable films for electrochromic devices, and nanomaterials for cancer therapy*.* The chemical formula of PBAs can be expressed as A_x_M_1_[M_2_(CN)_6_]_y_□_1-y_⋅zH_2_O, where A represents alkali or alkaline earth metals, and M_1_ and M_2_ typically denote transition metals interconnected by C≡N bonds forming a 3D open framework structure [66, 68, 69]. When PB and PBAs are used as cathode materials in SIBs, they may produce gas during battery operation. The causative factors of gas production mainly include the following aspects [23, 66, 68–70]: firstly, electrolyte decomposition. The structural stability of the Prussian blue cathode material is a key factor in hydrogen generation. Poor structural stability during charging and discharging leads to the decomposition of the electrolyte and generation of hydrogen. Water molecules in the Prussian blue cathode material also have a significant impact on hydrogen precipitation. It has been shown that the removal of interstitial water from Prussian blue cathode materials can significantly improve their performance as cathode materials for sodium-ion batteries. The stability of the electrolyte also affects hydrogen precipitation. Unstable electrolytes decompose under high pressure, producing hydrogen and other gases.

When Prussian blue is operated at high voltage, it will induce the solvents and salts in the electrolyte to decompose and produce gas. Secondly, water and impurities exist as side reactions. If water or other impurities exist inside the battery, they will react with the salts in the electrolyte or the cathode material to generate gases, such as water reacting with PF_6_^−^ to generate HF and POF_3_, structural changes in the cathode material and the formation and destruction of the SEI layer. Finally, overcharging can lead to the oxidation of the cathode material to produce oxygen, which is a common gas production phenomenon for PB and PBAs under overcharging conditions. As to PBAs, the gas evolution primarily stems from the trace crystalline water within the materials. As reactions proceed within the battery, inevitable voltage changes and temperature increases lead to breaking of HO–H bonds, resulting in the escape of OH^−^ and O atoms [66, 68, 70]. Particularly at elevated temperatures, adsorbed and lattice water within PBAs escape the crystal structure, entering the electrolyte and triggering side reactions that release gases such as H_2_O, NH_3_, and N_2_ [69–71]. Deng et al*.* [71] explored the gas production at the PW cathode, demonstrating H_2_ and CO_2_, respectively, and also found the possibility of HCN production. HCN is known to be highly toxic, which leads to the fact that the PW cathode will face more than just gas production leading to battery safety issues.

**Fig. 2:**
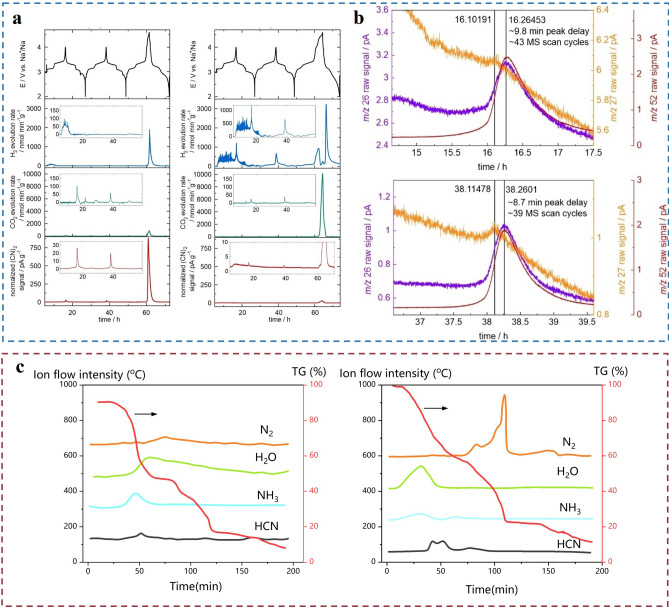
Gas production study on Prussian blue cathode materials. **a** Cathode gas production from Prussian blue cathode material. **b** Data support for demonstrating trace HCN production by PBAs during electrochemical processes. Reproduced with permission [71]. Copyright©2024 The Authors. Batteries Supercaps published by Wiley–VCH GmbH.** c** Four types of gases released in the range of 25–350 °C, H_2_O, NH_3_, N_2_, and HCN. During the decomposition of PB, both H_2_O and NH_3_ are released from PB-1H (left) and PB-3H (right). Thermal decomposition in the range of 480 to 600 °C produces N_2_, which indicates a complete disruption of the PB framework, resulting in the formation of a Fe/Fe_3_C composite. PB-3H releases a relatively large volume of toxic HCN in the temperature range from 230 to 320 °C, indicating the presence of H^+^ ions in the PB lattice. Reproduced with permission [23]. Copyright© 2020 Elsevier Ltd. All rights reserved

In order to eliminate the impact of water on gas production, You et al*.* synthesized high-quality Prussian blue (HQ-NaFe) nanocrystals ranging from 300 to 600 nm using Na_4_Fe(CN)_6_ as a single iron source precursor [70]. They pointed out that PBs prepared by conventional synthesis methods produce a large number of [Fe(CN)_6_] vacancies in the crystal framework due to the rapid precipitation process, and these vacancies are occupied by ligand water molecules. These water molecules decompose and produce gas during the charging process. Feng et al. investigated hierarchically structured hollow Prussian blue rods (R-PB) synthesized via sacrificial template methods as high-performance cathode materials for SIBs [69]. They mentioned that the water content can be reduced by removing interstitial water molecules in Na_2_MnFe(CN)_6_. With this dehydration treatment, the capacity of PBA was increased to ~ 150 mAh g^−1^ and the charge/discharge voltage plateau was flat at 3.5 V. Normally, the thermogravimetric analysis (TG) method is commonly applied for the water content calculation in the PBAs. However, Wang et al. present differing views on this matter [23]. They found that calculating PB’s water content based on weight loss within a specific temperature range (25–350 °C) is inaccurate. PB-1 h and PB-3 h (prepared in HCl solutions with different pH) released both H_2_O and NH_3_ within 25–350 °C, indicating the release of coordination water molecules from the PB lattice along with NH_3_ generation, indicative of hydrogen bond rupture with N in the framework. Furthermore, gas evolved between 480 and 600 °C predominantly composed of N_2_, signifying complete destruction of PB framework and formation of Fe/Fe_3_C composite materials. The thermal decomposition process shown in Fig. 2b for PB-1 h showed negligible release of toxic HCN gas; however, HCN was observed in PB-3 h between 230 and 320 °C, suggesting the presence of H^+^ ions within the PB lattice.

In addition to the decomposition reaction of water molecules that occurs when vacancies in the structure are occupied by ligand water molecules, the PB crystal structure may be distorted or collapsed during the insertion and extraction of sodium ions, particularly in the presence of a substantial number of [Fe(CN)_6_] vacancies [16], which can markedly enhance the structural instability.

### Polyanionic Electrode Materials

**Fig. 3:**
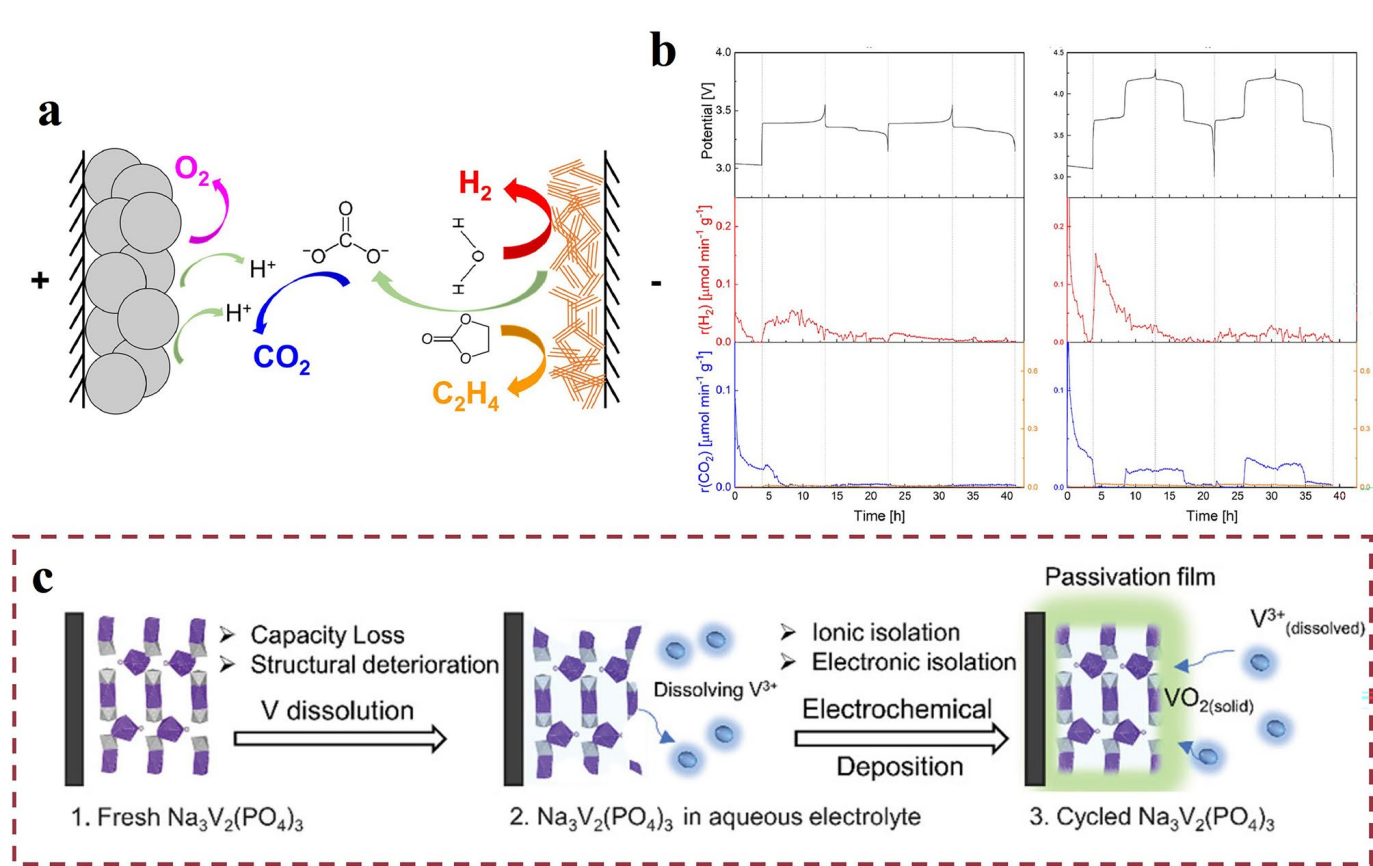
Mechanism of NVP decomposition and gas production. **a** Illustration of the decomposition mechanism of NVP. **b** Gassing of EC-based single-solvent electrolytes in NVP || Na (left), NVPF || Na (right). Reproduced with permission [72]. Copyright ©2021 The Author(s). Published by Elsevier B.V. **c** Degradation mechanism of Na_3_V_2_(PO_4_)_3_. Reproduced with permission [109]. Copyright © 2020 Advanced Energy Materials

Polyanionic electrode materials are favored over other types of cathode materials for their robust covalent bonding framework, high structural and thermal stability, enhanced cycle life and safety. They are widely considered as future Na-host electrodes. Despite the high safety, there is still a risk of gas production, Zhang et al*.* [72] hypothesized its gas production mechanism (Fig. 3a) and gas production was indeed detected in the vinyl carbonate electrolyte systems of NVP||Na and NVPF||Na (Fig. 3b). However, the intrinsic electronic conductivity of these materials is poor due to the presence of polyanionic units and the lack of direct M-OM-electron leaving domains. Zhang et al. emphasized the decomposition issues of electrolytes under high-voltage charging conditions when polyanionic compounds were employed as cathode materials in SIBs [73], including vanadium-based polyanionic compounds (Na_3_V_2_(PO_4_)_3_), iron-based polyanionic compounds (NaFePO_4_), manganese-based polyanionic compounds (Na_3_MnPO_4_CO_3_), NASICON-structured materials (Na_3_Fe_2_(PO_4_)_3_) [74], and mixed polyanionic compounds (Na_4_Fe_3_(PO_4_)_2_(P_2_O_7_)), respectively. Firstly, as a result of the electrolysis of water, H_2_ and O_2_ are produced at the cathode and anode, respectively. Secondly, vanadium-based polyanionic compounds (e.g., Na_3_V_2_(PO_4_)_3_) may dissolve in an aqueous electrolyte, giving rise to vanadium ions (V^n+^), which may react with hydroxide ions (OH^n−^) in the water to form vanadium hydroxide precipitates. Furthermore, Yue et al. also studied the degradation mechanism of Na_3_V_2_(PO_4_)_3_ (Fig. 3c). It corresponds to the previous statement that vanadium-based polyanionic compounds are soluble in aqueous electrolytes to produce vanadium ions. Finally, if the electrolyte contains acid ions, it may also decompose to produce gases such as SO_2_ and CO_2_. Dissolved gases in the electrolyte, especially oxygen, significantly impact the battery’s charge–discharge performance. Based on the aforementioned findings, they concluded the influence of different battery configurations on gas solubility and proposed electrolyte designs to control gas generation.

Na_2_FePO_4_F (NFPO) as a promising polyanionic cathode was extensively studied due to its excellent sodium-ion channels, structural stability, abundant iron ore resources, high theoretical capacity, and suitable operating voltage [49, 75]. Its polyanionic groups contribute to superior electrochemical performance but also undergo side reactions with electrolytes during electrochemical processes. First, for its special crystal structure, the crystal structure of NFPF contains channels for sodium-ion diffusion. If these channels are smoother, the de-embedding process of sodium ions will be smoother, reducing electrolyte decomposition and gas generation. Secondly, for its surface properties, the defects on the surface of NFPF (e.g., grain boundaries, vacancies, incomplete surfaces) act as active sites for electrolyte decomposition, which promotes electrolyte decomposition and increases gas generation. Finally, regarding its chemical stability, the redox potential of NFPF is about 3.2 to 3.8 V (*vs.* Na^+^/Na); if the potential is too high, it will lead to the oxidative decomposition of the electrolyte and gas generation. The potential variation of the cathode material during charge–discharge cycles leads to electrolyte decomposition at high potentials, forming a stable cathode CEI. Incompatible electrolytes exacerbate this decomposition, increasing battery resistance and decreasing performance and life span. Electrolyte decomposition typically involves the oxidation of solvents and electrolyte salts, particularly on the cathode material surface, resulting in the generation of various gases. Carbonate-based solvents such as ethylene carbonate (EC) and PC oxidize to produce CO_2_ at high potentials, while ester solvents and other additives generate O_2_ during oxidation. In some cases, oxidation of electrolyte additives or impurities like water molecules can also produce oxygen. Additionally, the use of fluorine-containing electrolyte salts like NaPF_6_ can decompose to generate hydrogen fluoride (HF) under high-temperature or overcharge conditions, causing severe damage to battery components, environmental concerns, and internal corrosion, thus affecting long-term stability and safety of the battery. The generation of these gases directly impacts the stability of polyanionic cathode materials and electrolyte compatibility.

In conclusion, when polyanionic compounds are used as cathode materials in SIBs, the decomposition and gas evolution voltage conditions pose complex challenges involving multiple chemical reactions and physical processes. The generation of these gases not only affects battery performance and life span but also poses safety risks. Therefore, a thorough understanding of the mechanisms behind these side reactions is crucial for developing safer and more efficient SIBs systems.

### Sulfur Cathode

Na–S batteries have attracted much attention due to their high energy density, high efficiency, and long life, but their performance is limited by the conductivity of the cathode material and the migration of polysulfides. Sulfur, as the cathode material of Na–S batteries, is prone to react with components in the electrolyte during charging and discharging, leading to gas generation [76–79]. For example, sulfur will react with impurities or decomposition products in the electrolyte to generate gases such as H_2_S. Xiong et al*.* focused on gas-related probes in Na_2_S cathode materials in room-temperature sodium–sulfur (RT-Na/S) batteries [76]. They detected the generation of H_2_, CO, and CO_2_ gases during the first sodium replenishment of the study samples by the DEMS technique and showed that these gases originated from the decomposition of the carbonate electrolyte. In addition, they utilized an ultrasound scanning technique to study the gas generation process inside the Na_2_S-4||Cu button cell. The blue areas in the ultrasound images indicate low ultrasound transmittance, indicative of the gas generation behavior.

Moreover, the shuttle effect of polysulfides during charging and discharging can lead to decomposition of the electrolyte and gas generation. The generation of gases is further exacerbated by the migration of polysulfides in the electrolyte and their reaction with the electrode materials. By designing sulfur-based cathode materials with unique structures, such as nanostructured sulfur@carbon composites, Lu et al*.* can effectively improve electrochemical performance and reduce gas generation [79]. For example, embedding sulfur nanoparticles into highly layered and spongy carbon matrix can shorten the diffusion path of Na^+^ and accelerate electron transfer. This work improves the electrochemical performance and cycling stability of room-temperature sodium–sulfur batteries by indirectly reducing the unfavorable reactions that may lead to gas generation through the optimization of electrolyte solvent and cathode materials.

Currently, there are relatively few studies on gas generation from cathode materials in Na–S batteries, and more advanced technological means are needed to detect and analyze the gas generation process. On-line electrochemical mass spectrometry (OEMS) and other techniques can provide more detailed gas composition and generation mechanisms for the study. It is also possible to optimize the composition and additives of the electrolyte, which can reduce the side reactions between the electrolyte and the cathode material, thus reducing the gas generation. At the same time, modification of the cathode–electrolyte interface is also an important means to improve the performance of Na–S batteries.

## Gas Evolution from Anode Materials

The gas evolution mechanism of anode materials in SIBs primarily involves chemical reactions during the charge–discharge processes, particularly interactions between the anode materials and electrolyte. In SIBs, various common anode materials undergo side reactions during cycling, leading to gas generation. These reactions include: (1) Formation of a stable SEI on the anode surface during initial charging is accompanied by gas evolution; (2) solvents in the electrolyte decompose on the anode surface during charging, producing gases; and (3) interactions between the anode materials and certain components in the electrolyte also generate gases. These gas evolution phenomena significantly impact battery performance and life span.

### Carbon-Based Anode Materials

Graphite is a common anode material in LIBs, offering moderate lithium storage capacity (approximately 350 mAh g^−1^) compared to Li^+^/Li. However, research indicates that graphite does not intercalate sodium ions effectively [80, 81]. Non-graphite anodes, primarily composed of various carbonaceous materials such as carbon black and pitch-based carbon fibers [82, 83], allow for sodium-ion insertion and are considered the "first-generation" anode choices for SIB systems. Carbon-based anodes mainly include hard carbon (HC) and soft carbon. HC, widely used as an anode electrode material for SIBs, exhibits high specific capacity (exceeding 300 mAh g^−1^) and a low potential platform (typically below 0.1 V *vs*. Na^+^/Na).

**Fig. 4:**
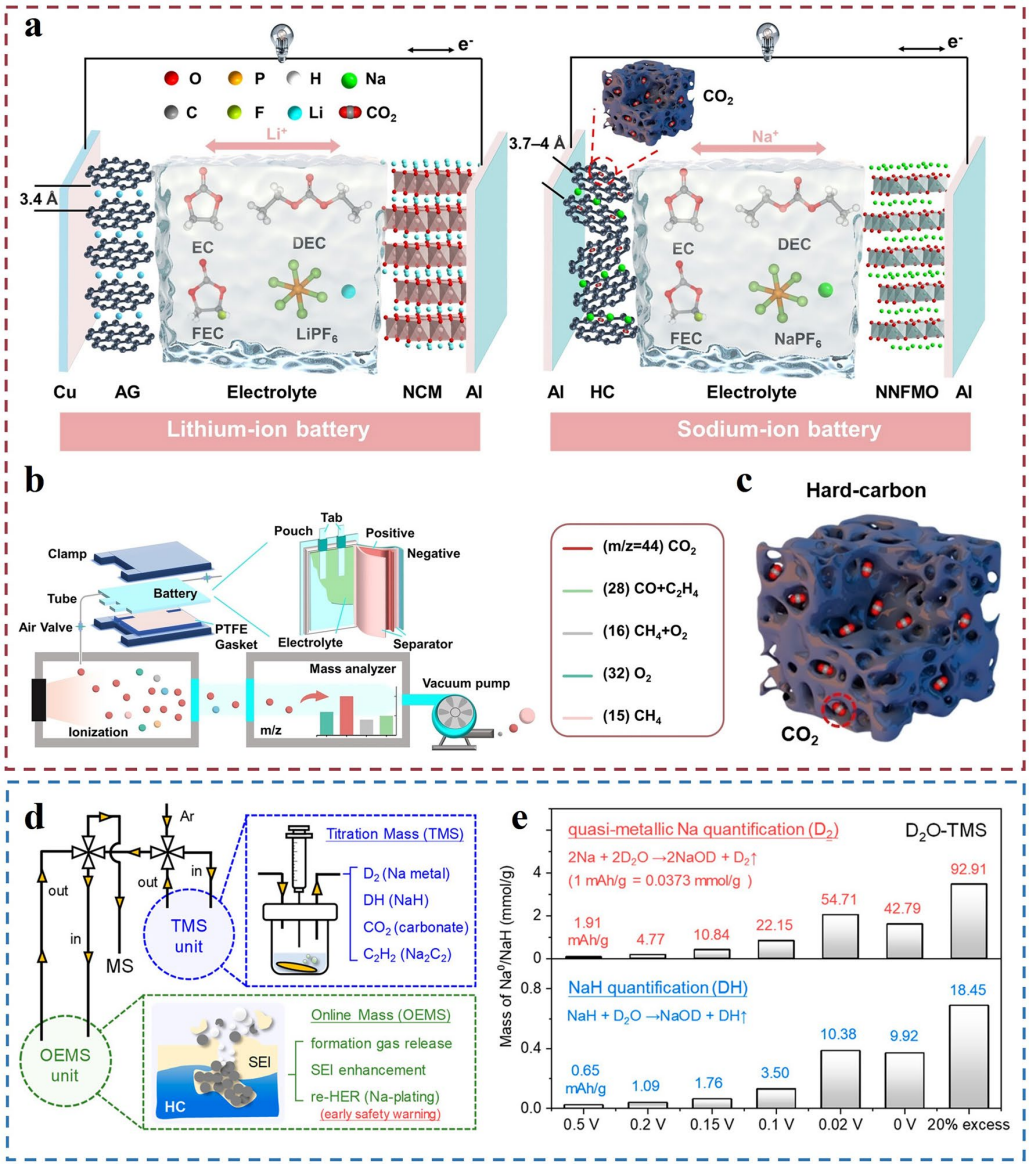
Gas evolution behavior of HC anodes and associated gas analysis results. **a** Schematic diagram of gas production during the operation of LIBs and SIBs under the condition of using HC materials at the same time. **b** Schematic diagram of DEMS test setup using sodium pouch battery under HC material. **c** Schematic diagram of the adsorption mechanism of HC materials for carbon dioxide gas. Reproduced with permission [87]. Copyright © 2024 Wiley–VCH GmbH **d** Schematic diagram of the TMS/OEMS gas analysis system. The TMS device is used for quantitative analysis. The OEMS device is used for real-time monitoring of the gas release behavior. **e** D₂O-based TMS results for HC electrodes at different discharge cutoff voltages (0.5, 0.2, 0.15, 0.1, 0.02, and 0 V, 20% overdose) are shown. Reproduced with permission [90]. Copyright © 2024, American chemical society

HC [84], as an advanced electrode material, has garnered significant attention in battery technology due to its unique physical and chemical properties. A critical factor influencing its performance is the pore structure, which comprises both open pores (open porosity) and closed pores (closed porosity), together forming the porous nature of HC. This porous structure provides numerous active sites for the electrolyte, thereby increasing the contact area between the HC and the electrolyte. During the charge–discharge cycles of a battery, the electrolyte undergoes decomposition at these active sites, resulting in gas generation [51]. The high specific surface area of HC further enhances this contact, significantly increasing the likelihood of electrolyte decomposition and gas release. This gas generation not only affects the battery’s performance but also poses potential risks to battery safety and longevity [21]. Specifically, the gases generated from electrolyte decomposition include various types. For instance, carbonate solvents produce CO_2_ upon decomposition, while proton sources such as water molecules generate H_2_ [22, 83, 85]. In organic solvents, the cleavage of C–H bonds can lead to CH_4_ formation. Under extreme conditions, such as battery overheating or overcharging, CO_2_ may even decompose further into CO [86]. A detailed and in-depth study [87] of the gas adsorption and desorption behavior of HC [88, 89] anodes during charging and discharging have been done by Deng et al*.* They compared the principled difference between lithium and sodium, and revealed the mechanism of adsorption of CO_2_ gas on HC anode during charging and desorption during discharging (Fig. 3a–c). This study explains the gas release phenomenon using an adsorption–desorption mechanism. It also emphasizes that adsorption and desorption of gases affect the formation and stability of the SEI layer. Adsorbed CO_2_ interferes with the uniform growth of the SEI layer, while desorbed CO_2_ leads to poor contact between the electrolyte and electrode interface. Finally, an effective solution—introduction of 5A molecular sieves—is proposed. Overall, this study reveals the effects of adsorption and desorption behaviors of gases in SIBs on the SEI formation process through an in-depth study, and proposes an effective solution to improve the battery performance. Similarly, the study of Xue et al*.* combined OEMS and TMS techniques (Fig. 4d) to reveal the sodium storage behavior and gas release mechanism of HC anodes in sodium-ion batteries [90]. And the results showed (Fig. 4e) that small amounts of Na metal and NaH were detected at 0.5 V, and the amount of these compounds gradually increased with the discharge process.

Furthermore, defects in HC significantly impact battery performance. Edge defects, vacancies, and heteroatom defects (e.g., oxygen, sulfur, phosphorus, nitrogen, and boron) in HC provide additional storage sites for sodium ions, thereby enhancing the sodium storage capacity of HC. However, the presence of oxygen and phosphorus doping in carbonate solvents can promote the generation of CO_2_ from decomposition products. These structural features not only alter the electronic structure of HC but also affect its interactions with the electrolyte, thereby influencing the charge–discharge performance and cycle stability of the battery. Therefore, in-depth research into the pore structure, defect types, and their impact on battery performance is crucial for developing high-performance, high-safety battery systems. By optimizing the preparation process and structural design of HC, the generation of gas can be effectively controlled, thereby improving the overall performance and life span of the battery.

### Sodium Metal Anodes

Sodium metal anodes (NMAs) are pivotal components in full-cell systems, offering high energy densities. NMAs provide a high theoretical capacity of 1166 mAh g^−1^ and a low redox potential of 2.71 V *vs*. SHE. However, NMA materials face stability issues including unstable SEI in organic electrolytes, dendritic growth, and gas evolution. During the initial charging of sodium metal batteries, the NMA contacts the electrolyte, initiating the formation of SEI. This process involves the decomposition of electrolyte components on the anode surface, leading to gas evolution as a side reaction. For example, Chen et al. conducted detailed research on the decomposition mechanisms of electrolytes on NMA materials and the generation of gases [46]. They concluded that sodium (Na) is more prone to gas evolution in ionic solutions, compared to being more pronounced in pure solvents. This difference was attributed to the fact that the lowest unoccupied molecular orbital (LUMO) energy level of the complex formed by the ion with the solvent was lower than that of the pure solvent. The lower LUMO energy level means that the complex is more likely to be involved in a chemical reaction, which leads to more gas production. In short, the presence of ions lowers the LUMO energy of the solvent complex, making gas evolution reactions more likely to occur.

**Fig. 5:**
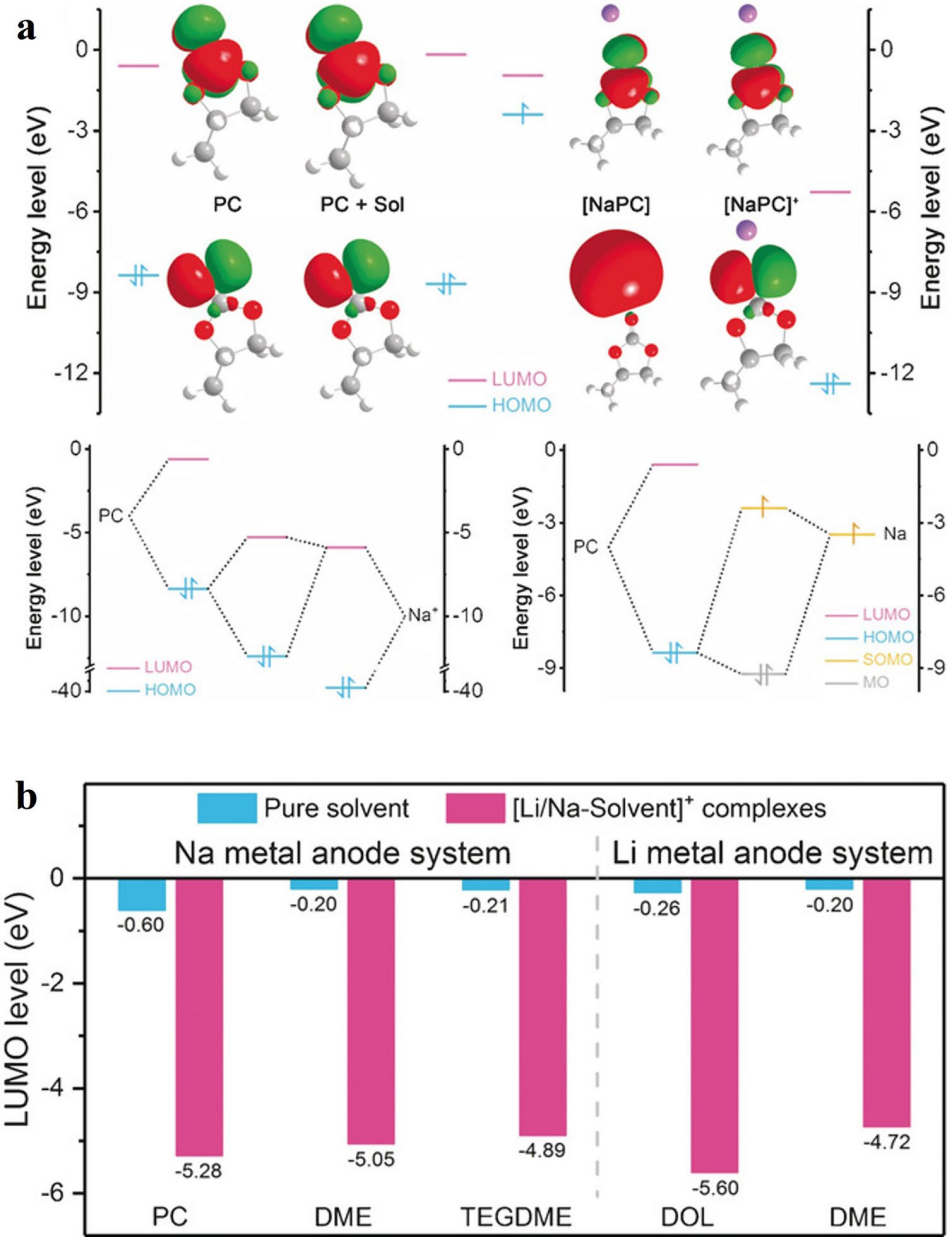
Comparison of orbital hybridization schematic and LUMO levels. **a** The upper panel shows the levels of frontier molecular orbitals for PC (single PC molecule), PC + Sol (PC molecule with solvent effect taken into account), [Na–PC] (Na atom–PC complex) and [NaPC]^+^ (Na^+^ –PC complex). The red and green regions represent the positive and negative parts of the LUMO and HOMO wave functions, respectively (equivalence: 0.02). In addition, hydrogen, lithium, carbon, and oxygen atoms are labeled in white, purple, gray, and red, respectively. The lower left panel shows the schematic diagram of orbital hybridization between PC and Na^+^, and the lower right panel shows the schematic diagram of orbital hybridization between PC and Na atoms. Reproduced with permission [46]. **b** Comparisons among the LUMO levels of pure solvents and ion–solvent complexes in Na and Li metal anode systems. Reproduced with permission [46] Copyright © 2018 Angew. Chem. Int. Ed.

Further insights into gas evolution mechanisms were provided through AIMD (ab initio molecular dynamics) simulations, which generally apply to various electrolyte (e.g., PC, 1,2-dimethoxyethane (DME), tetra ethylene glycol dimethyl ether (TEGDME), 1,3-dioxolane (DOL))–metal (e.g., Na, Li) systems [46]. When PC is used as a solvent, complexes formed between sodium ions (Na^+^) and PC significantly lower the LUMO energy to 5.28 eV upon complexation (Fig. 5a, b), indicating enhanced reducibility of [Na–PC] complexes on sodium metal, thereby triggering gas evolution [46]. The energetic difference between PC and [Na–PC] is as high as 5.11 eV, explaining the hypothesis that ion coordination weakens solvent molecule stability and promotes gas generation. Gases produced include carbon monoxide and propylene, both of which are flammable, thereby increasing safety risks.

**Fig. 6:**
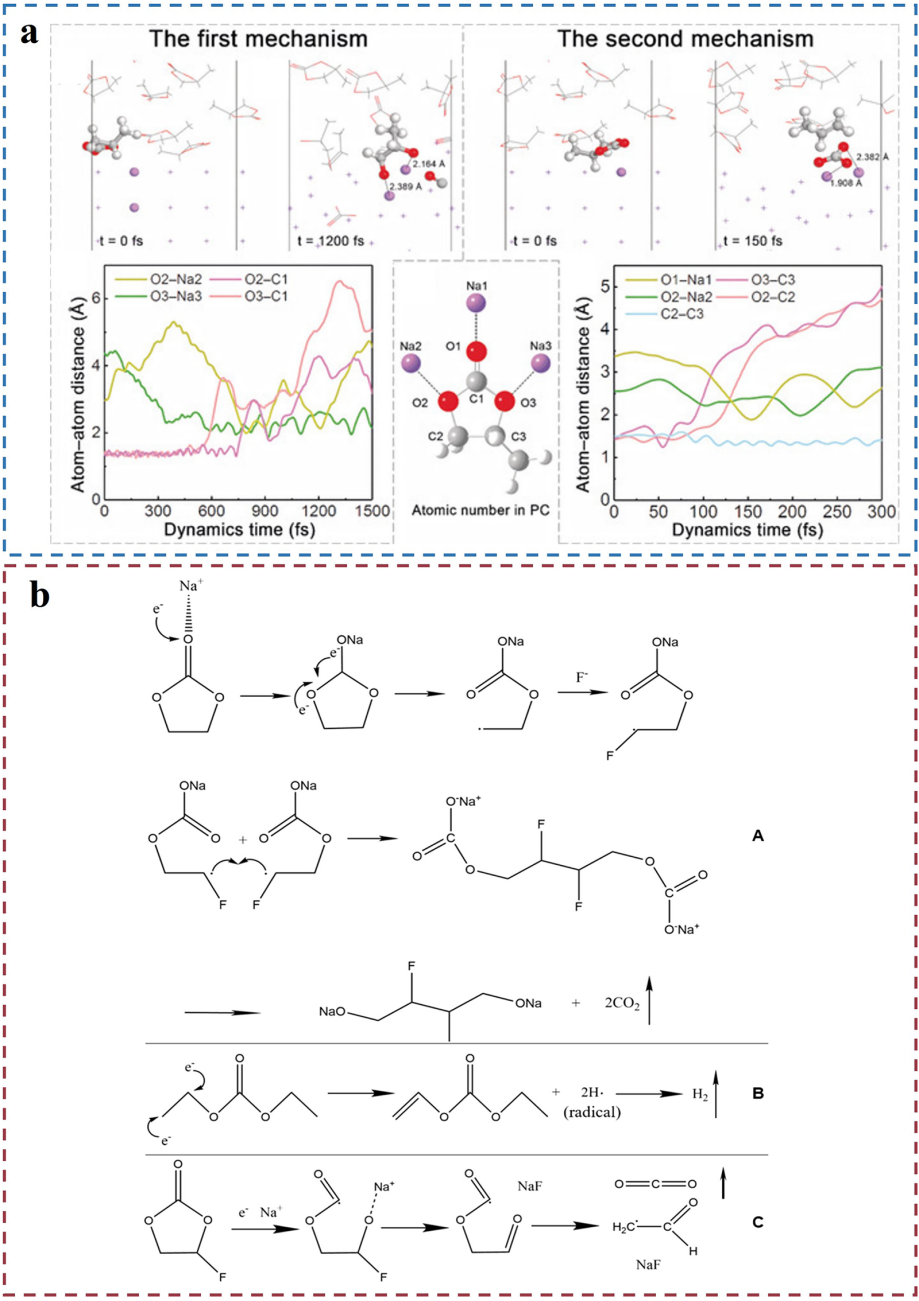
AIMD simulation results and decomposition gas production mechanism of different solvents. **a** AIMD was used to simulate the two decomposition mechanisms of PC molecules on the Na (110) surface at different times (upper panel) and the time evolution of the whole process (lower panel). The hydrogen, lithium, carbon, and oxygen atoms are labeled in white, purple, gray, and red, respectively. Only the reacting PC and Na atoms are shown in a ball-and-stick model. The other atoms are shown as lines. Reproduced with permission [46]. Copyright © 2018 Angew. Chem. Int. Ed. **b** Possible decomposition gas production mechanisms for (A) EC, (B) dimethyl carbonate (DEC), and (C) fluoroethylene carbonate (FEC). Reproduced with permission [51]. Copyright © 2019, American Chemical Society

Moreover, through AIMD simulations (Fig. 6a), a mechanism of electrolyte decomposition involving carbon monoxide generation was proposed. In this mechanism, a PC molecule is reduced on the sodium metal surface, leading to the formation of carbon monoxide and organic sodium salts. This process involves electron transfer from PC molecules, breaking the C-O bond and subsequently releasing a carbon monoxide molecule [46]. Additionally, sodium metal reacts with carbonate-based solvents such as EC, PC, DEC, and DMC to produce corresponding sodium salts and gases (Fig. 6b). The general form of these reactions can be represented as: 2ROCO_2_^−^ + Na → Na_2_CO_3_ + CO_2_↑ + R_2_, where RO- represents the alkoxyl group of the solvent and R denotes the hydrocarbon chain [51]. Furthermore, Chen’s group [46] mentioned that even when using ether-based solvents like DME and TEGDME, gas generation still occurs in sodium metal batteries.

### Alloy-Type Anodes

Alloy-type anode electrode materials refer to materials formed by metallurgical alloying of sodium metal ions with alloying elements such as tin (Sn), antimony (Sb), and indium (In) for SIBs. These materials typically exhibit high theoretical specific capacities. For instance, tin (Sn) has a theoretical capacity of up to 847 mAh g^−1^, which is advantageous for enhancing the energy density of batteries. Furthermore, alloying reactions often occur at lower potentials, implying that alloy anode electrode materials can provide a stable potential platform during the charge–discharge processes of SIBs, thereby enhancing overall battery performance.

However, alloy-type anode electrode materials also face challenges in SIBs [91]. These challenges include capacity degradation after prolonged cycling and, more critically, issues such as inhomogeneous sodium insertion during the alloying process and volume fluctuations. The localized expansion of volume can result in the formation of stress concentrations within the material, potentially leading to cracking and structural damage over extended cycling periods. Structural damage, particularly when cracks and fractures occur, exposes fresh surfaces that can undergo chemical reactions with the electrolyte, resulting in gas generation. Additionally, during charging, a significant number of gases (e.g., CO_2_) are adsorbed by the anode material and released upon discharge as its adsorption capacity diminishes. Simultaneously, due to volume expansion and structural damage, the SEI membrane is continuously ruptured and reformed, accompanied by gas production throughout this process [43]. Therefore, the fundamental issue remains as electrolyte decomposition caused by structural damage and increased side reactions. Despite the structural irreversibility of the alloying process, it is important to design the alloying process more rationally for the side reaction challenges of batteries at high open-circuit voltage.

## Gas Evolution from Electrolytes

Electrolytes play a crucial role in SIBs, not only influencing the electrochemical performance but also directly impacting the safety and longevity of the batteries. Firstly, electrolytes serve as the medium for sodium-ion migration between the cathode and anode electrodes, determining the battery's conductivity and ion transport efficiency [82]. An excellent electrolyte should possess high ionic conductivity, good chemical stability, and a wide electrochemical window. Secondly, the stability of the electrolyte directly affects the battery's cycling stability and operational life span. If electrolyte decomposition occurs during battery charge and discharge cycles, it can generate gases and solid by-products, thereby reducing the battery's energy density and posing safety risks such as short circuits, thermal runaway, and even explosions [47, 71]. Therefore, systematically summarizing the phenomena and mechanisms of electrolyte decomposition and gas generation is crucial for the future development of high-performance, long-life, and safe SIBs.

### Organic Electrolyte

The utilization of organic electrolytes provides a broader electrochemical range, enhanced conductivity, and superior battery performance. Consequently, there is a substantial market acceptance for battery technologies based on organic electrolytes, with an already well-established supply chain and recycling system in place. Organic electrolyte systems employ organic solvents (such as carbonates: EC and EMC) blended with sodium salts (e.g., NaPF_6_, and NaBF_4_) and additives. The selection of additives for sodium batteries can best follow some strategies that can both enhance stability and inhibit gas production. For example, selecting film-forming additives to hinder the reaction between electrode materials and electrolyte; combining multiple additives (e.g., molecular and ionic additives) to improve the mechanical strength of the interfacial film and enhance the stability of the film; and selecting additives that are easy to be decomposed quickly and do not produce gas (e.g., per fluorobenzene, Na_2_C_2_O_4_, and NaDFOB) [77]. Moreover, in order to form a stable SEI/CEI layer, additives with high reducing activity (FEC, VC, etc*.*) can be selected, which can preferentially reduce the negative electrode surface to form an SEI layer enriched with stabilizing components, such as NaF, and thus effectively inhibit the decomposition of the electrolyte [92]. In addition, the introduction of inorganic salt additives (NaI, Na_2_CO_3_, etc*.*) is also an option. In order to reduce the decomposition of the electrolyte, solvents and additives that are not easy to decompose and additives that will not decompose within the operating voltage range of the battery should be selected, and the water and impurity content of the electrolyte should be controlled.

**Fig. 7:**
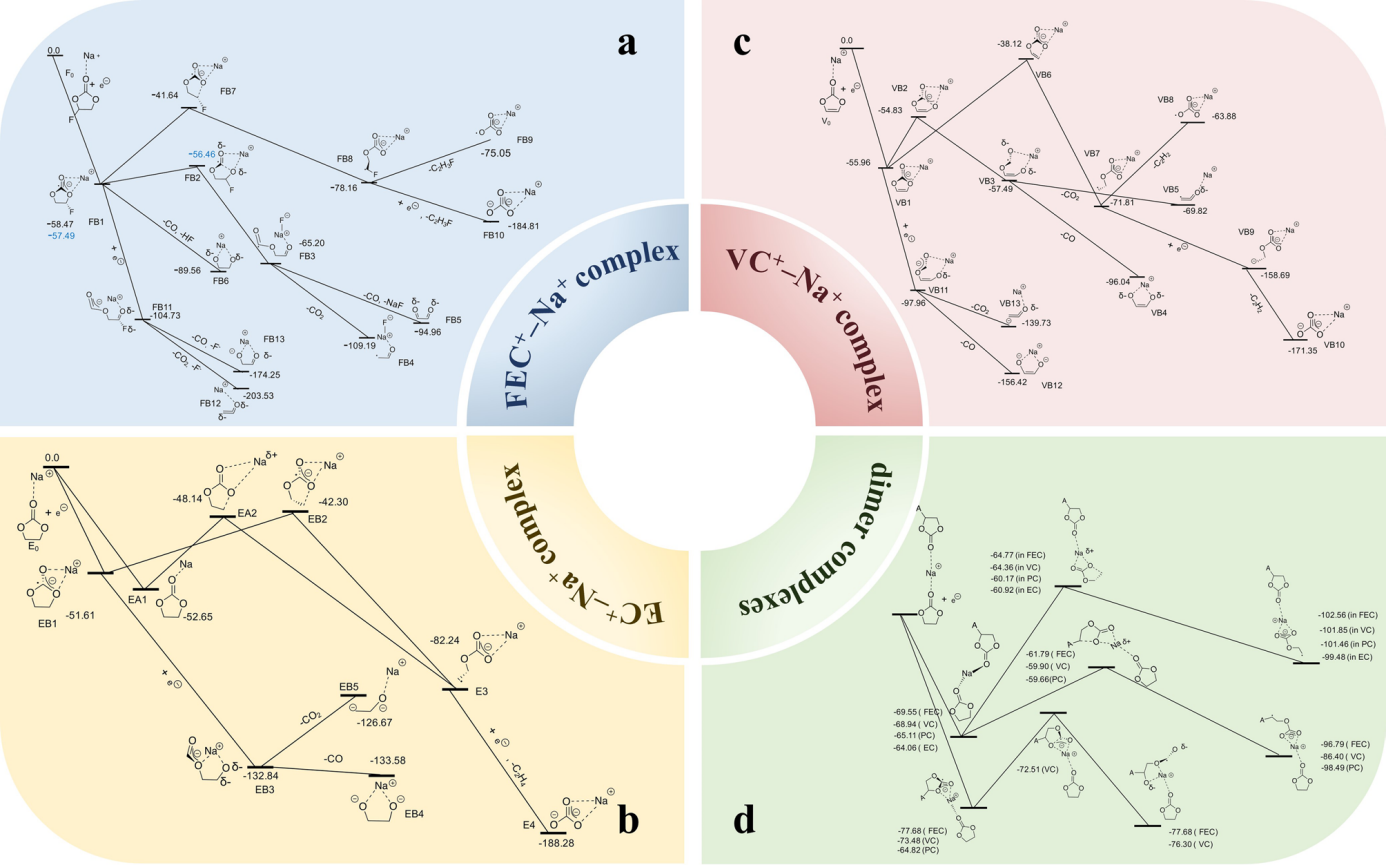
Specific pathways of the reductive decomposition reactions of various complexes have been investigated using the continuum medium model (PCM). (T = 298.15 K and p = l atm.). Reproduced with permission [22]. Copyright © 2016, American Chemical Society

Normally, during initial battery testing, solvent and salt decomposition on the anode surface form SEI film. A comprehensive study on the formation of SEI film was conducted by Kumar’s group [22]. They found that additive molecules have significantly lower decomposition barriers, thus decomposing first. And the presence of additive molecules increases the decomposition barrier of EC (Fig. 7a). In the two-electron reduction process of EC, dissociation of the CEOC bond leads to the release of CO_2_ gas molecules, while forming a -CH_2_CH_2_O- anion (EB5). The Gibbs free energy change (ΔG) for this process is 6.17 kcal mol^−1^. Meanwhile, in the two-electron reduction process of EC, dissociation of the CCOC bond also releases CO gas and forms a -OCH_2_CH_2_O- anion (EB4). The ΔG for this process is − 0.78 kcal mol^−1^. During the decomposition of FEC in Fig. 7b dissociation of the C–CO-C bond also releases CO_2_ gas and generates a CHCHO- anion (FB +). The ΔG for this process is − 43.99 kcal mol^−1^, indicating it is thermodynamically favorable. In FEC decomposition, dissociation of the CCOC bond releases CO gas and forms a CH_2_CHO_2_- anion (FB5). The ΔG for this process is − 29.76 kcal mol^−1^. In the decomposition of vinylene carbonate (VC) in Fig. 7c, dissociation of the CCOC bond results in the release of CO gas and formation of a -OCHCHO-anion (VB4). The ΔG for this process is -38.55 kcal mol^−1^. The reaction pathways discussed above lead to the formation of anionic radicals, and of the many different reaction possibilities, the attack of excess electrons on anionic radicals to generate carbonate anions and release ethylene molecules is the most prominent one. The thermodynamic data corresponding to this reaction in the presence of the additive molecule is shown in Fig. 7d.

In sodium-ion batteries, high open-circuit voltage does lead to electrolyte decomposition, due to the reduced chemical stability of the electrolyte at high voltage, which makes it susceptible to decomposition reactions. The thermal stability of the electrolyte is critical to battery safety. At high voltages, the electrolyte may undergo violent decomposition and produce hazardous substances, which will continue to erode the positive electrode, leading to severe capacity degradation of the battery. In order to increase the operating voltage of sodium-ion batteries, electrolytes need to be designed to withstand high voltages. Commercially available NaPF_6_/carbonate-based electrolytes exhibit severe oxidative decomposition at operating voltages greater than 4.2 V, forming unstable SEI/CEI on the surface of the positive and negative electrodes, leading to low Coulombic efficiency and accompanied by the generation of hazardous substances, which can severely corrode the SEI/CEI and the materials, accelerating the degradation of the battery. But in addition, studies have shown that as the internal temperature of SIBs rises, a series of chemical reactions occur inside the battery, releasing a large amount of heat and gases. These reactions include salt decomposition and reaction with solvents to generate new SEI, SEI membrane decomposition reaction, electrolyte evaporation, etc*.*

In traditional carbonate-based electrolytes, chemical and electrochemical decomposition on sodium metal surfaces produce olefins, alkanes, CO, and H_2_ gases [50]. These flammable gases pose significant safety risks in practical applications. Furthermore, continuous gas evolution (such as olefins, CO, and CO_2_) and by-products of in situ cell generation (primarily Na_2_CO_3_) disrupt the stability of the SEI, leading to continued dendrite growth and very low Coulombic efficiency (CE). Lee et al. performed gas analysis of the electrochemical processes in sodium metal batteries using different electrolyte systems through in situ DEMS [93]. By observing gas phenomena and detecting by-products, they inferred gas generation reactions, including the chemical decomposition of PC producing Na_2_CO_3_ and C_3_H_6_, and the decomposition of FEC producing CH_2_CHO and CO_2_.

**Fig. 8:**
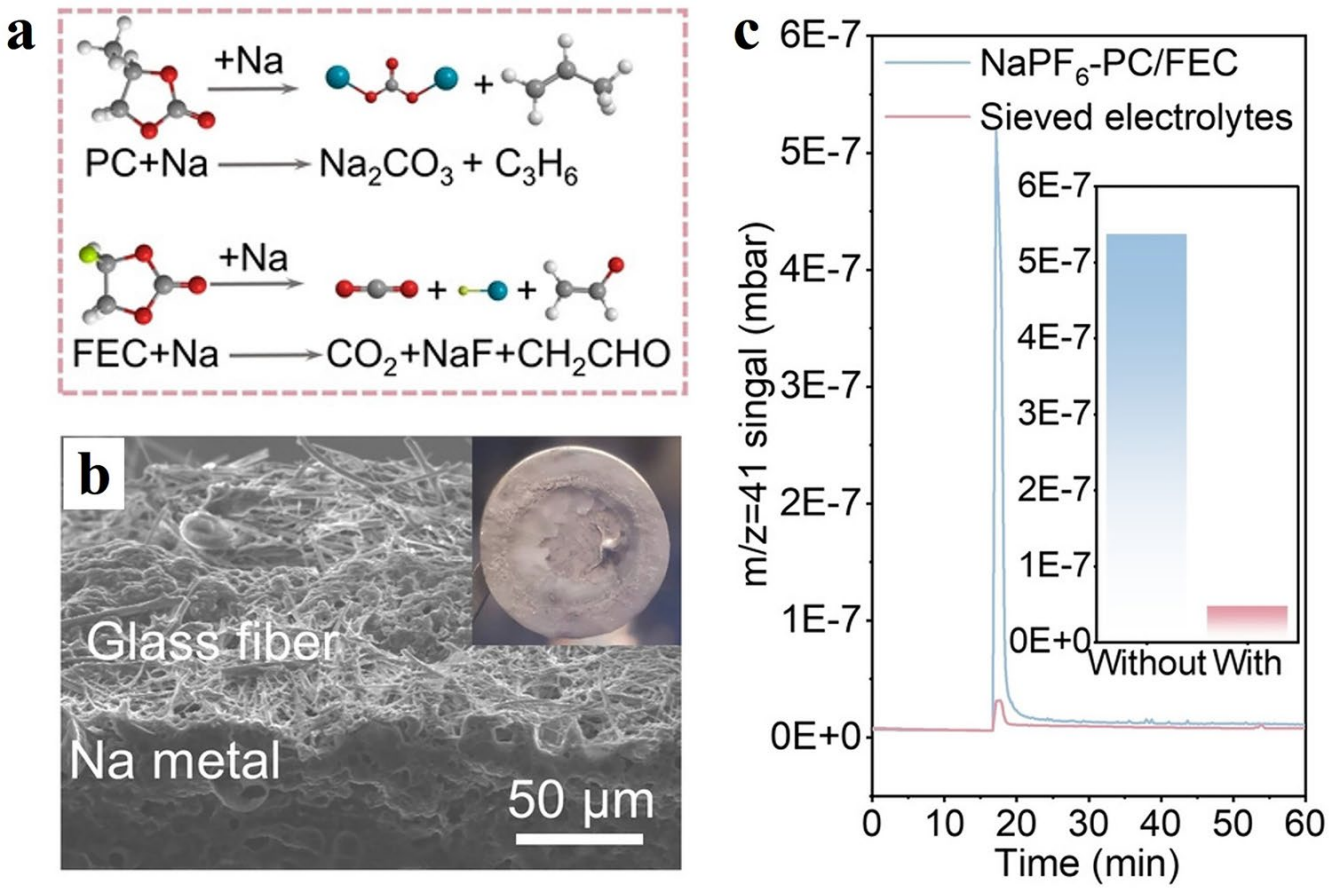
Reaction speculation and results. **a** Reactions for hypothesized gas evolution. **b** Heavily corroded fiberglass film. **c** Quantification of gas evolution in two electrolytes by gas chromatography. Reproduced with permission [50] Copyright © 2022, Angew. Chem. Int. Ed.

As the most important parts of gas production, CO_2_ and CO have received the most attention in the study of gas evolution for SIBS. Lu et al. [50] deduced the side reactions produced by the gas by observing the gas phenomena and detected by-products, including the chemical decomposition of PC to produce Na_2_CO_3_ and C_3_H_6_, and the decomposition of FEC to produce CH_2_CHO and CO_2_ (Fig. 8a). In addition, due to the reaction of the by-products produced by the accompanying gas with the silica-aluminum oxides in the diaphragm, the glass fiber diaphragm is severely corroded and large holes appear, which are penetrated by sodium dendrites (Fig. 8b). The evolution of C_3_H_6_ and CO_2_ was quantitatively analyzed using gas chromatography (GC) in the literature, and it was found that the gas production of C_3_H_6_ and CO_2_ in the screened electrolyte was significantly lower than that in the NaPF_6_–PC/FEC electrolyte (Fig. 8c).

**Fig. 9:**
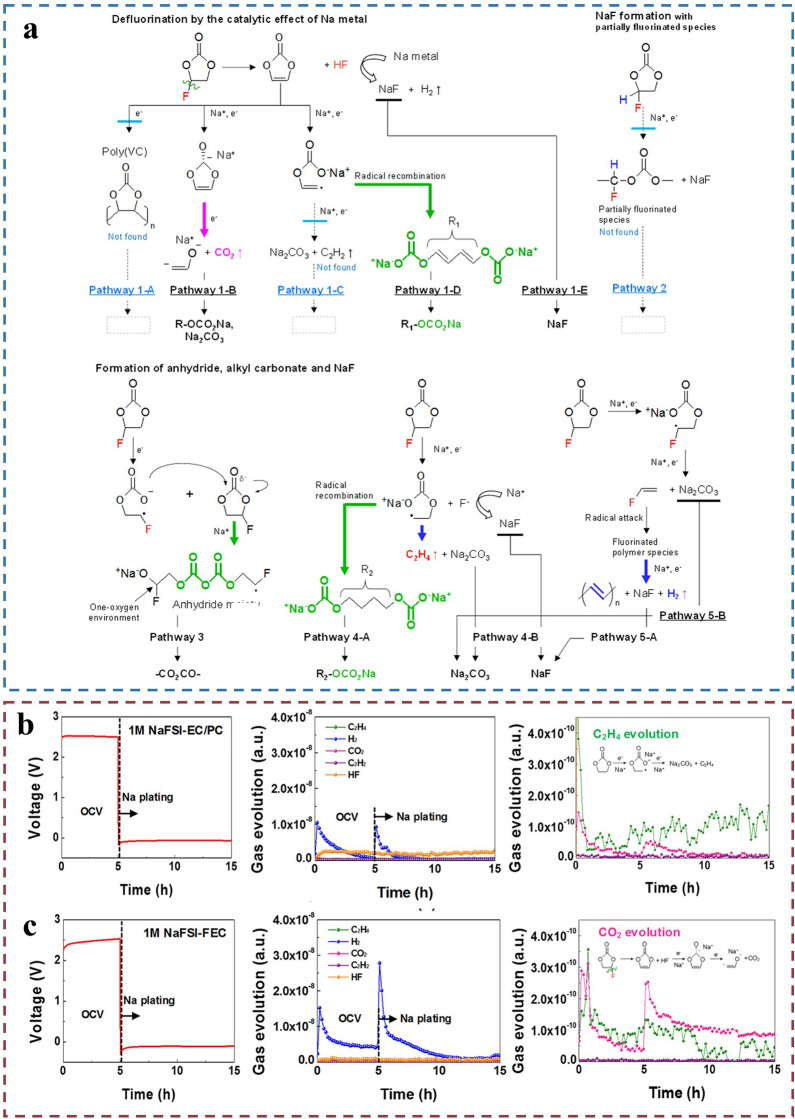
Gas generation mechanisms and data from in situ transmission electron microscopy and in situ DEMS. **a** Possible mechanisms for the construction of the FEC-derived interlayer. Reproduced with permission [72]. Copyright © 2021, Energy Storage Mater. **b** In situ DEMS of gas precipitation in Na /electrolyte/ Cu cell structures. Voltage profiles and in situ DEMS data of C_2_H_4_, H_2_, CO_2_, C_2_H_2_, and HF gases in 1 M NaFSI-EC/PC (1/1). **c** 1 M NaFSI-FEC initially plated Na/Cu cells at 0.282 mA cm^−2^ at 25 °C. Reproduced with permission [93]. Copyright © 2018, American Chemical Society

There is currently a great deal of interest in studies targeting FEC decomposition mechanisms. They presented various pathways for FEC decomposition resulting in the formation of compounds such as R-OCO_2_Na, Na_2_CO_3_, and NaF, and its decomposition produces various gases, including C_2_H_4_, H_2_, CO_2_, C_2_H_2_, and HF (Fig. 9a). The CO_2_ signal detected by DEMS indicates that the decomposition of FEC molecules contributes to CO_2_ formation through pathway 1-B. Previous studies have highlighted the adverse effects of CO_2_ produced by electrolyte decomposition on batteries [72]. However, Lee et al*.* [93] proposed a novel conclusion that the various gases generated from FEC decomposition, especially a moderate amount of CO_2_ **(**Fig. 9b, c), help in forming a carbonate-based SEI layer. This enhances the safety and cycling stability of the battery.

### Solid-State Electrolyte

Solid-state batteries, utilizing solid-state electrolytes (SSIs), offer higher energy density and improved safety and are thus considered promising alternatives to liquid electrolyte systems. The three main types of solid-state electrolytes currently under investigation are solid polymer electrolytes (SPEs), composite polymer electrolytes (CPEs), and inorganic solid electrolytes (ISEs). These electrolytes play a crucial role in solid-state SIBs. The obvious advantages of solid-state batteries over liquid batteries include high energy density and high safety. However, solid-state batteries with high energy density also face some problems, such as interfacial stability. High energy density usually requires the use of high-voltage cathode and high-capacity anode materials, which can increase the interfacial instability between the electrodes and the solid-state electrolyte. It is the interfacial instability within the battery that can cause serious gas production problems. It is well known that solid-state electrolytes and solid-state electrolyte interfaces can effectively mitigate gas evolution in metal-ion batteries [94]. As a protective barrier in SIBs, the SEI provides essential functions of electronic insulation and ionic conduction, preventing electrolyte decomposition and electrode material corrosion. This, in turn, enhances the coulombic efficiency and cycling stability of the battery, significantly impacting its overall performance and life span. A well-formed SEI layer helps reduce side reactions during charge and discharge cycles. In LIBs, the SEI layer also prevents lithium dendrite formation, which is equally important for SIBs, despite the lower tendency of sodium metal to form dendrites [4, 95]. However, gas production problems can occur even in solid-state batteries.

In solid-state SIBs, inorganic solid electrolytes (ISEs) such as Na–β/β″-alumina and NASICON, with their high ionic conductivity and excellent mechanical strength, help suppress sodium dendrite growth, thereby enhancing battery safety. However, these materials can react with sodium metal, leading to gas release, such as the decomposition of sulfide electrolytes in air or humid environments, producing H_2_S gas [54, 55, 96]. Sulfide electrolytes, such as many sulfur-containing solid electrolytes, typically exhibit poor stability in air or humid environments. This is due to their tendency to react with water molecules or oxygen in the environment, leading to electrolyte decomposition and the production of hydrogen sulfide (H_2_S) gas and other by-products. The authors [55] explored the air stability of the sulfide-based solid electrolyte Na_3_SbS_4_. This material was designed with the hard and soft acid and base (HSAB) theory in mind to improve its stability under ambient conditions. The HSAB theory states that hard acids tend to react with hard bases, while soft acids tend to react with soft bases. In the context of sulfide electrolytes, sulfur (S^2−^) is usually considered a soft base, while many metal ions, such as phosphorus (P), are considered hard acids. Under ambient conditions, a hard base (e.g., oxygen) may react with a hard acid (e.g., phosphorus), displacing the soft base (e.g., sulfur) and causing the electrolyte to decompose. This reaction usually produces H_2_S gas as follows: Metal_n+_S + O^2^ → Metal_n+_O + S_2_^2−^ and S_2_^2−^ + H_2_O → H_2_S. In the literature, authors have noted that most phosphorus-containing superionic conductors of phosphorothioates (e.g., Na_3_PS_4_) are unstable in air because they readily react with oxygen to produce H_2_S gas.

### Others

In sodium batteries, polymer electrolytes and quasi-solid electrolytes each have an important scientific position. Polymer electrolytes are suitable for flexible batteries and application scenarios requiring high safety due to their solid-state properties, high safety and good mechanical properties. However, their room-temperature ionic conductivity is low. And despite their relatively high safety, they are not immune to internal gassing behavior, where the polymer electrolyte undergoes electrochemical decomposition at high voltages, generating gases. For example, polyethylene oxide (PEO)-based electrolytes decompose to produce gases such as H_2_ and CO_2_ when the voltage exceeds their electrochemical stability window. Also, at high temperatures, polymer electrolytes undergo thermal decomposition, releasing gases. For example, PEO also decomposes to form CO_2_ at high temperatures.

In addition, the quasi-solid electrolyte combines the advantages of solid and liquid electrolytes, has a high ionic conductivity and good mechanical properties, and is suitable for high-magnification and long-life applications, and has a good application prospect. However, its ionic conductivity at room temperature is still low. And the interfacial side reaction between the quasi-solid electrolyte and the sodium metal negative electrode leads to a continuous increase in interfacial resistance. This side reaction usually involves the reaction of the liquid components of the electrolyte with the sodium metal to form unstable intermediates (e.g., Na_2_CO_3_ and NaF) These intermediate contribute greatly to the occurrence of both gas-producing side reactions. At high voltages or high temperatures, the liquid components in the quasi-solid electrolyte are prone to decomposition or even volatilization, generating gases and solid by-products, which not only increase the interfacial resistance, but also lead to electrolyte failure or even battery explosion.

## Methods to Suppress Gas Generation

### Suppression of Gas Generation in Cathode Materials

#### Structural Doping of Cathode Materials

Among the current cathode materials for SIBs, layered transition metal oxides are the most commercially successful due to their high theoretical capacity and abundant sodium reserves, presenting promising application prospects. Do et al. conducted a comparative study of sodium-rich layered transition metal oxides and concluded that ordered O3-type materials exhibit better structural stability [40]. However, rapid capacity decay and severe safety issues caused by detrimental phase transitions and significant volume strain greatly limit their practical applications. Doping of layered transition metal oxides can mitigate the structural rearrangement caused by sodium-ion insertion and extraction in SIBs, thereby reducing oxygen loss during charging and suppressing gas evolution [30, 41, 97, 98].

For instance, Yu et al*.* made considerable efforts to stabilize the internal crystal structure of layered transition metal oxides and control harmful phase transitions and volume strain [99]. They introduced light boron doping in interstitial positions, which reduced the energy gap between P2 and O3 structure formation energies, leading to the formation of P2 and O3 biphasic structures at high Na states. Additionally, due to the lattice interlocking effect of P2 and O3, the biphasic structure exhibited nearly zero volume strain. The stabilization of the structure reduces side reactions between the cathode material and the electrolyte, which reduces the generation of gases. During battery charging and discharging, the instability of the cathode material leads to a reaction with the electrolyte, generating gases, such as oxygen or hydrogen. Since the volume strain of the biphasic structure is almost zero, the volume change of the battery during charging and discharging is effectively controlled, which reduces the change in the contact area between the active material and the electrolyte due to volume expansion, thus reducing the possibility of gas production. The stabilization of the structure helps to improve the cycling stability of the battery. During the battery cycling process, the continuous volume expansion and contraction leads to the destruction of the material structure, and the introduction of doping elements reduces the possibility of such destruction, thus improving the cycle life of the battery. Due to the stabilization of the structure of the cathode material, the possibility of electrolyte decomposition is reduced, thus reducing the amount of gas produced due to electrolyte decomposition.

**Fig. 10:**
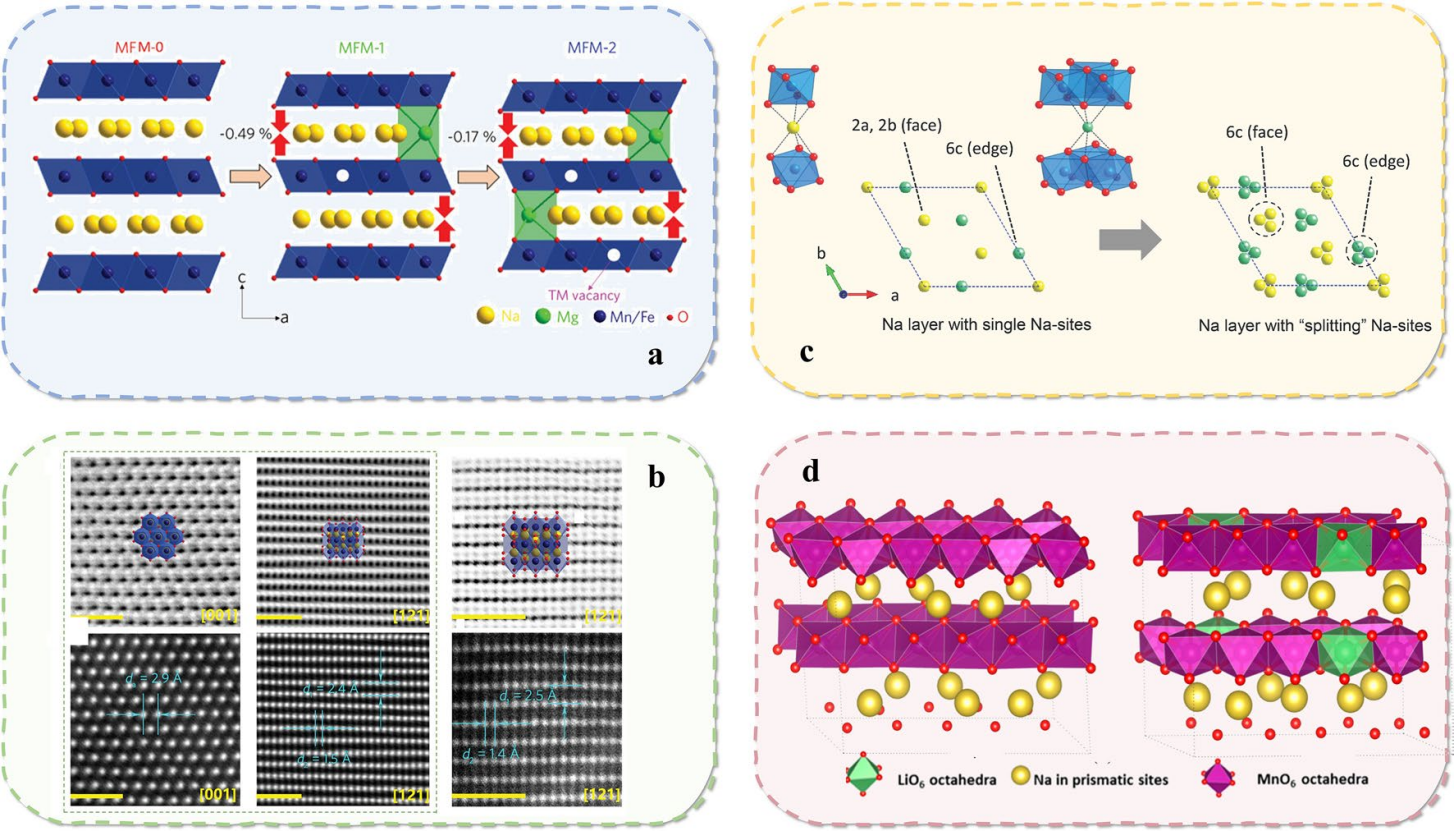
Internal structure optimization of sodium cathode materials. **a** Crystallographic evolution of MFM-x (x = 0, 0.1, 0.2) after Mg substitution for vacancies in P2 layered Na_0.7_Mg_x_[Fe_0.4-x_Mn_0.6_□_x_]O_2_. The lower panel shows the local coordination environment of Na with the vacancy above Na in the TM layer. The non-bonded oxygen 2*p* orbitals pointing to the vacancy position are depicted on the right [60]. **b** STEM technique was utilized to accurately identify the structural evolution during Na extraction and insertion. The scale bar in each panel represents 1 nm. The lower panel is a schematic representation of the observation directions along the [001] and [121] axes, respectively. Reproduced with permission [60]. Copyright © 2021, J. Mater. Chem. A. **c** P2-type Na_5/6_[Li_1/4_Mn_3/4_]O_2_ schematic, as well as calculated electron densities for the sodium layer, and shows two different crystal structure sites, Li-rich and Mn-rich sites, found in the TM layer. Reproduced with permission [30]. **d** P2-type structures based on a 5 × 2 supercell of hexagonal NaMnO_2_; (a) Na_0.6_MnO_2_; (b) Na_0.6_Mn_0.8_Li_0.2_O_2_. Reproduced with permission [30]. Copyright © 2021, Adv. Mater

Li et al*.* successfully introduced natural vacancies in the transition metal positions of P2 layered cathode materials by incorporating Mg^2+^ into the Na layer [60]. The prepared Na_0.7_Mg_0.2_[Fe_0.2_Mn_0.6_□_0.2_]O_2_ cathode with inherent transition metal vacancies demonstrated superior cycling stability and rate performance. The non-bonding 2*p* orbitals of oxygen pointed toward the TM vacancies, theoretically triggering reversible oxygen redox reactions over a wide voltage range of 1.5 to 4.5 V. By forming TM vacancies in the original P2 layered cathode, oxygen redox was activated at the beginning of the charging process. Within this voltage range, the P2 phase maintained its structure well **(**Fig. 10a, b), even at high current rates of up to 10 C. The study revealed the charge compensation mechanism of MFM-2, where Mn^3.5+^/^4.0+^ and O^2−^/^−^ redox contributed to the entire voltage range of 1.5 to 4.5 V, and Fe^3+^/^3.5+^ redox mainly contributed to the high-voltage region of 3.0 to 4.5 V. Mg ions in the Na sites acted as "pillars," preventing structural collapse along the c-axis during charging and effectively suppressing oxygen release at high voltages. Additional studies [30] of the crystal structure of layered cathode materials of the P2 type are shown in Fig. 10c, d. These findings provide new opportunities for designing reversible oxygen redox cathode materials with high structural stability.

Through in situ XRD analysis, Zhu et al*.* confirmed that copper doping enhances the stability of PBAs during charging and discharging. This implies that copper doping helps to maintain the structural integrity of the material and reduce gas production due to structural damage [100]. The co-doping of Cu, Co, and Ni, especially the introduction of Cu as a pillar supporting the PBA framework, can significantly improve the structural stability of the material. The improved structural stability helps to reduce the phase transition and volume expansion during charge/discharge cycling, both of which are the main causes of gas production.

There have been many studies on structural modifications to reduce structural phase transitions to minimize structural damage and the occurrence of gas production side reactions. The in situ XRD testing of CuCoNi111 and CuCoNi011 elucidated the electrochemical insertion/extraction mechanism. During sodium-ion extraction from CuCoNi111, there was initially no significant change in the lattice, maintaining a perfect cubic Fd-3 m space group. However, when the voltage rose to around 3.0 V, a new peak near 38.5° appeared, which could be indexed as (331) and included in the PDF card of the Fm-3 m lattice parameter. This transition indicated that CuCoNi111 experienced minimal phase transition during extraction, maintaining its cubic structure. During reduction, with sodium ions reinserted into the CuCoNi111 sample, the (331) peak gradually diminished. Eventually, the crystal structure returned to its original state, indicating that the lattice parameter changes in CuCoNi111 were highly reversible during cycling. Therefore, with Cu doping, CuCoNi111 exhibited minimal volume change during charge–discharge cycles, thereby protecting the CuCoNi-based PBA lattice framework. Overall, Cu-doped CuCoNi111 exhibits small volume changes and highly reversible lattice parameter changes during charge/discharge cycling, which contributes to the reduction of outgassing and improves the performance and safety of SIBs.

#### Surface Coating of Cathode Material

Phase transitions from layered to spinel structures, lattice disruption and pore formation are the main factors leading to cycle capacity degradation in metal-ion batteries and, more critically, to mechanical strain, structural deformation and even collapse, which can lead to serious safety issues such as gas generation inside the battery. Researchers generally believe that surface coatings or chemical additives can partially stabilize the structure. These methods can reduce side reactions between materials and electrolyte, enhance structural stability, and improve battery cycle life and safety. The cladding methods include: chemical vapor deposition (CVD), which generates a solid film through the chemical reaction of gaseous reactants on the surface of the material; physical vapor deposition (PVD), which forms a thin film on the surface of the substrate by transferring the material from the target material to the substrate through physical methods (e.g., evaporation, sputtering); and atomic layer deposition (ALD), a self-limiting vapor deposition technique in which a single atomic layer is precisely controlled by alternating the introduction of different reactive gases; sol–gel, in which a coating is formed on the surface of a material by the chemical processes of sol–gel and gel; calcination, in which the material is heated to a high temperature, resulting in physical and chemical bonding between particles to form a dense coating or structure; and electrochemical deposition, in which a coating or structure is formed by electrochemical reactions. In electrochemical deposition, a specific material is deposited on the surface of a conductive material through an electrochemical reaction. Coating materials include Al_2_O_3_, AlF_3_, TiO_2_, nitrides (e.g., Si_3_N_4_), carbon materials (e.g., graphene, carbon nanotubes), and polymer coatings (e.g., polyvinylidene fluoride (PVDF)). As early as 2013, Gu et al. applied an AlF_3_ coating to metal-ion batteries and found that it could partially alleviate the formation of spinel within the layered structure during cycling, thereby reducing side reactions and mitigating capacity decay [101]. However, they also found that the AlF_3_ coating could not completely prevent spinel formation.

In the context of SIBs, P2-type Na_2/3_[Ni_1/3_Mn_2/3_]O_2_ has been considered a promising cathode material due to its high theoretical capacity (173 mAh g^−1^) and long operational voltage plateau (4.2 V). Liu et al. compared the pristine and cycled Na_2/3_[Ni_1/3_Mn_2/3_]O_2_ samples and discovered that the delamination of transition metal oxide layers and crystal phase transitions were the main reasons for gas production side reactions and capacity decay [102]. To improve cycling performance and battery safety, the researchers applied an Al_2_O_3_ coating, which effectively suppressed unfavorable side reactions and delamination of the metal oxide layers at high voltage. The Al_2_O_3_ surface coating not only reduced side reactions at high voltage but also provided mechanical support, helping to maintain the layered structure and enhance the reversibility of the P2-O2-P2 phase transition. This unlocked stable cycling performance of the P2-type layered structure materials within a high-voltage window and consequently suppressed gas evolution.

### Suppression of Gas Generation in the Electrolyte

The decomposition of electrolytes has consistently been the primary pathway for internal gas generation in SIBs, particularly within electrolyte systems. Various research teams have extensively studied and analyzed different electrolyte systems. Lu et al. conducted research on gas evolution in various SIBs with different electrolyte systems, focusing particularly on CO_2_ and other flammable gases [50]. The research team used gas chromatography (GC) for the quantitative analysis of C_3_H_6_ and CO_2_ evolution. They found that the gas production of C_3_H_6_ and CO_2_ in the screened electrolytes was significantly lower than that in the original NaPF_6_–PC/FEC electrolyte. Furthermore, under battery abuse conditions, such as over-discharge, batteries using the screened electrolyte demonstrated a higher average coulombic efficiency compared to those using the original NaPF_6_–PC/FEC electrolyte (99.6% vs. 98.1%), indicating that the screened electrolyte has a robust capability to suppress gas generation and reduce safety risks.

In addition to the self-decomposition of electrolytes under high-temperature or process conditions, the deposition of sodium metal in different electrolytes leading to significant gas generation and fragile porous dendrite issues is also very severe. Rodriguez et al. explored the morphology of sodium metal deposition and gas generation in different electrolytes, focusing specifically on the impact of FEC as an electrolyte additive [103]. The research team found that sodium deposition in EC, DEC, and PC produced a large amount of gas and fragile porous dendrites** (**Fig. 10a). In the experiments, when FEC was used as a co-solvent to mix with DEC, CO_2_ production was reduced, but still present. This suggests that even in FEC/DEC electrolytes, where large amounts of gas and labile porous dendrites are produced, the decomposition of carbonate species is also a major source of gas production. In addition, oxygen production can be observed throughout the high-voltage charging process. This is related to the release of lattice oxygen due to the instability of the oxide cathode material in the high charge state. Comparison of multiple electrolyte systems shows that the co-solvent of FEC and DEC (1:1 v/v) exhibits better cycling performance, impedance, and cycling efficiency when FEC is used, although porous deposition still exists, and also significantly reduces the gas generation during the deposition process.

The screening and optimization of electrolytes for SIBs are a complex process involving multiple consideration. In addition to the direct screening of different electrolytes, the optimization of the electrolyte structure can also be used to achieve improved safety. For example, Xia et al*.* [104] introduced ether-based solvents to improve the performance of electrolytes. They showed that NaDFOB-based ether electrolytes exhibit excellent electrochemical stability, solvent compatibility, and the ability to form a dense, robust SEI on the electrode surface. In addition, additives can be used appropriately. Li et al. [105] proposed a novel anion receptor additive, 4-aminobenzeneboronic acid pinacol ester (ABAPE), for attenuating the coupling between anions and cations and accelerating the transport kinetics of Na^+^. ABAPE formed hydrogen bonding with H_2_O/HF, which effectively prevented hydrolysis and stabilized the acidic species of NaPF6.

Moreover, research on quasi-solid-state SIBs is also very hot in order to take safety into account. Luo et al*.* are at the forefront of this research [88]. They proposed a novel triangular synergistic strategy to accelerate the conduction of sodium ions through the interactions between polymer–salt, ionic liquid, and electron-rich additives. This strategy fully utilizes the advantages of each component to achieve fast ion transport and stable battery performance. With this strategy, the researchers succeeded in improving the ionic conductivity of the polymer electrolyte to 1.37 × 10^–3^ S cm^−1^, which is a significantly higher value than that of conventional polymer electrolytes. Meanwhile, the controlled decomposition of NaTFSI salts led to the formation of a thin SEI layer enriched with organic fluorine, which facilitated the rapid transport of sodium ions and improved the interfacial stability of the cell. All of these studies provide new directions for the development of new high-safety and high-energy-density battery systems.

### Suppression of Gas Generation in Anode Materials

**Fig. 11:**
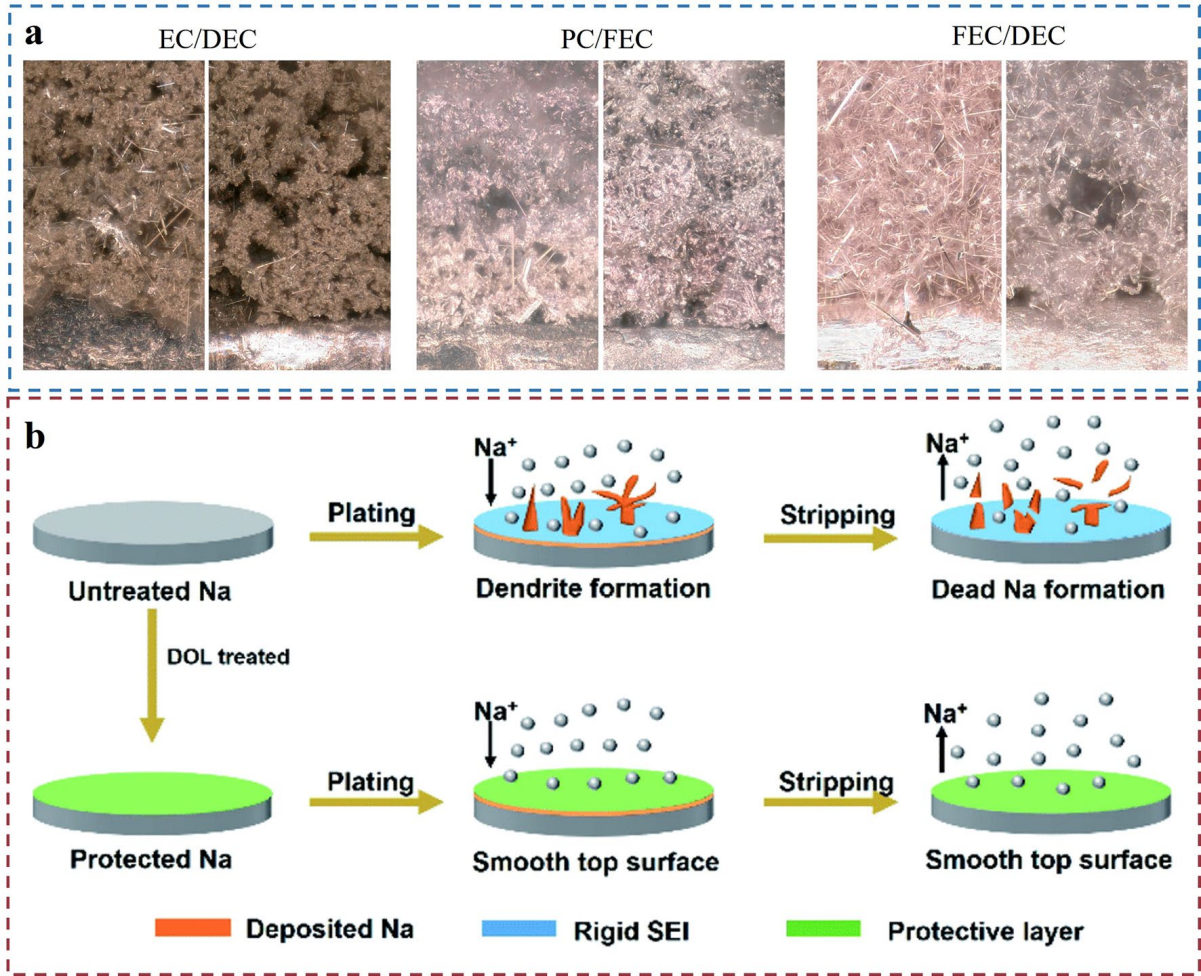
Schematic deposition status and behavior. **a** Morphologies of sodium deposited from 1 M NaPF_6_ in EC/DEC, PC/FEC, and FEC/DEC upon passage of 4 mAh cm^−2^ at 1 mA cm^–2^ (top images) and 5 mA cm^–2^ (bottom images). Reproduced with permission [103]. Copyright © 2017, ACS Energy Lett. **b** Schematic illustration of the Na plating and stripping behavior of untreated (uppercase) and protected Na (lowercase). Reproduced with permission [106]. Copyright © 2017, American Chemical Society

As mentioned in the discussion of cathode materials, scientists are now keen to utilize surface coatings to control the growth of dendrites, thereby reducing the potential for side reactions between the cathode material and the electrolyte. This approach is not only applicable to cathode materials but also extensively researched in other areas of SIBs for performance optimization through surface coatings. For instance, Lu et al*.* investigated a simple method to stabilize sodium metal anodes to enhance sodium battery performance [106] (Fig. 11b). They highlighted the extreme and uncontrollable dendrite growth and gas evolution issues with sodium metal anodes, which lead to low Coulombic efficiency and severe safety hazards such as dangerous short circuits and even explosions. The research team developed an effective protective layer on the sodium metal anode through a simple pretreatment method using DOL. The protected sodium electrodes exhibited excellent electrochemical performance in symmetric Na–Na cells. In full cells, batteries with protected sodium electrodes demonstrated significantly improved cycling stability compared to untreated sodium electrodes. The spray coating technique successfully demonstrated the potential scalability of the protection strategy, making the transition from coin cells to 20 cm^2^ pouch cells feasible.

Testing conditions (such as temperature, voltage, and current density), as well as the optimization of the synthesis process for electrode materials and electrolytes, are crucial for suppressing gas evolution during electrochemical operations. By optimizing the electrode materials, optimizing the electrolyte, and accurately controlling the test conditions such as temperature, voltage, and current density, it is possible to reduce the side reactions during battery operation and thus reduce the gas production. The most critical of these is the optimization of electrodes materials and electrodes, and the screening and optimization of electrodes are discussed in detail next. For instance, Chen et al. prepared nitrogen-doped porous carbon nanofibers (CNFs) and carbon nanotubes (CNTs) using bimetallic organic framework (MOF)-based composites through self-etching and graphitization methods [107]. When synthesizing nitrogen-doped carbon materials on an industrial scale, parameters such as yield scaling, controlled atmosphere, temperature gradient, humidity control, and calcination protocols must be precisely controlled to ensure the structural stability and properties of the materials while reducing unnecessary gas generation. Optimization of these parameters is critical to achieving an efficient, economical and environmentally friendly production process. Their optimization of electrode materials helps suppress gas release side reactions during electrochemical processes The synthesized nitrogen-doped carbon nanotube paper (CHT paper) exhibited ultra-long cycling life (over 10,000 cycles) and high rate performance, with a reversible capacity of up to 346 mAh g ^−1^ in SIBs. This structural optimization helps to reduce the side reactions during charging and discharging, thus reducing the gas production. Similarly, Xu et al. emphasized that selecting the appropriate carbonization temperature and protective atmosphere is key to preparing high-performance HC [31]. Under different preparation atmospheres, the electrochemical performance and specific surface area of the HC materials will be very different, which directly affects the gas production of the cell. The electrochemical performance of HC materials varied significantly depending on the atmosphere in which they were prepared, primarily due to differences in specific surface area. In the preparation of HC materials, the selection of a suitable protective atmosphere (e.g., nitrogen or argon) reduces the oxidation or reduction of the material surface and thus improves the electrochemical properties of the material. Although these gases primarily affect the material synthesis process, they have a significant impact on the final properties of the material and its stability during battery operation. Overall, although gases in the sintering atmosphere are not generated directly from within the cell, the properties of the electrode materials can be improved by optimizing the synthesis temperature, thus indirectly reducing gas generation during cell operation. All of the above optimizations contribute to the safety and cycle stability of the battery.

## Conclusion and Perspective

This paper provides a comprehensive and unified understanding of gas generation mechanisms in SIBs, encompassing cathode materials, anode materials, and electrolytes. Specifically, the gas generation in cathode materials is primarily attributed to their instability under high-voltage conditions. For instance, during the insertion and extraction of sodium ions, cathode materials may undergo phase transitions or decomposition processes, resulting in the release of oxygen or other gases. Anode materials can generate gases through side reactions with the electrolyte during charge and discharge cycles. Moreover, electrolytes can also decompose at high temperatures or high voltages, leading to gas production. Additionally, components such as conductive carbon black and residual water can also serve as sources of gas during battery operation.

In response to the aforementioned gas generation mechanisms, we have summarized several strategies for suppressing gas formation. First of all, as the most important component inside the battery, the screening, optimization, and appropriate additives of the electrolyte are all very helpful to inhibit gas production. Meanwhile, setting a buffer layer between the electrode material and electrolyte can also effectively reduce the direct contact between them, thus reducing the occurrence of side reactions. Secondly, optimization and enhancement of electrode materials, such as the development of more stable cathode and anode materials, can significantly minimize gas production. Additionally, designing rational compositions for electrodes structures, along with adjusting synthesis conditions (such as temperature and pressure), can also aid in suppressing gas generation.

Gas detection and analysis are fundamental to addressing the gas generation issue in SIBs. However, currently widely adopted equipment like DEMS has several limitations, including restricted detection duration due to electrolyte evaporation, deviations from real battery systems caused by the use of large amounts of electrolyte, and inaccuracies in the collected gas production results. Therefore, further enhancements in DEMS equipment are necessary and imperative. Obtaining compelling gas production results enables more precise conclusions regarding gas generation mechanisms. This not only facilitates the analysis of the root causes of battery degradation but also provides accurate and targeted strategies for suppressing gas formation. Based on the aforementioned summary, the future evolution mechanism and inhibition approaches of gas in SIBs are proposed:

**Optimization of the battery system**. Firstly, the stability of the electrolyte has a direct impact on the gas production of the battery during the charging and discharging process of SIBs. Gas production can be reduced by developing new electrolytes. Electrolyte additives can change the gas production behavior of SIBs during cycling, which will be an effective means to prepare high-performance, long-cycle SIBs. Therefore, the development of new electrolytes and additives to improve the chemical stability and electrochemical window of the electrolyte is one of the key ways to reduce gas production. Secondly, the choice of positive and negative electrode materials also has a significant impact on the gas production phenomenon. It is found that the best commercialized laminated cathode material has higher energy density, but also has the fatal disadvantage that it is more likely to lead to the generation of H_2_ and CO_2_, while the polyanionic cathode material shows superior electrochemical performance. Therefore, gas production can be effectively reduced by optimizing cathode materials, such as developing materials with more stable structures that are insensitive to ambient humidity. Finally, improving the stability of the electrode–electrolyte interface is also an important strategy to reduce gas production. Improving the stability of the electrode–electrolyte interface in SIBs through interfacial coating modification can reduce the side reactions, thus reducing gas production. Meanwhile, the sodium compensation strategy can optimize the full-cell performance and reduce gas production due to insufficient sodium ions. The initial coulombic efficiency of the battery can be improved and gas production can be reduced through the presodiumation process.

**Battery environment management**. Firstly, temperature management, the performance of SIBs will be affected under wide temperature range conditions, and the change of temperature will significantly affect the chemical reaction and reaction rate within the battery, which in turn will have an important impact on the formation of the electrode/electrolyte interface, the safety performance of the battery, its service life, its stability, and various types of electrochemical characteristics. During the assembly process, the ambient humidity must be strictly controlled to prevent the material from absorbing moisture, and dust-free or low-dust conditions must be ensured to avoid the mixing of impurities. Appropriate temperature control helps maintain material stability, while proper pressure application ensures close contact between the electrodes and electrolyte. The application of automation and precision assembly technology helps to improve the consistency and reliability of the battery. In addition, considering the operating characteristics of SIBs at different temperatures, the battery management system (BMS) needs to be specially designed to accommodate their unique voltage characteristics and over-discharge tolerance. Combining these factors, an optimized assembly environment can enhance the performance and safety of SIBs in a wide range of temperatures to meet the future needs of electric vehicles, energy storage, and other applications.

**Introduction of other high-resolution means.** Future research should focus on developing multifunctional in situ DEMS devices with high-precision, long-term gas detection capabilities. Such devices should possess capabilities for extended testing durations, advanced volatile electrolyte condensation and replenishment systems, highly sensitive gas detection abilities, and standardized and reproducible gas testing protocols. In commercial battery testing, pouching phenomena typically occur after several hundred cycles, well beyond the current capabilities of DEMS. Therefore, there is a need for devices capable of long-term testing to perform in situ gas generation analysis over extended cycling processes. Some electrolytes may be carried away by vacuum systems or carrier gases, which can shorten battery life and clog capillaries, thereby affecting device sensitivity. Hence, condensation systems are required to capture volatile electrolytes, thereby reducing interference with instrument sensitivity and test results. Furthermore, in long-term gas generation studies of SIBs, internal gas consumption needs to be considered for accurate analysis. Typically, the gas produced by DEMS batteries is limited, and quantitative results from different batches are difficult to replicate, making standardized testing protocols crucial and worthy of promotion.

Future efforts also need to focus on developing precise detection technologies to maximize gas information acquisition and deepen understanding of gas generation mechanisms. In situ DEMS can provide information on gas generation initiation points and gas content, complemented by other techniques detecting solid and liquid reaction products to estimate gas generation mechanisms. Various gas suppression methods are needed to address multiple sources of gas in SIBs. Modifying and optimizing all potential gas generation components before battery assembly can implement multiple measures while suppressing gas production. The mechanism of gas production and the suppression method under different working conditions are studied deeply. In-depth studies on gas generation mechanisms and suppression methods under normal and thermal runaway conditions can simultaneously enhance the electrochemical performance and safety of SIBs. Gas analysis is crucial for achieving high safety and electrochemical performance in SIBs and requires more attention and research in the future. Through systematic research and optimization, important scientific basis and technical support can be provided for the development of efficient and safe SIBs. This not only contributes to advancing the commercialization of SIBs but also provides more reliable energy solutions for large-scale energy storage and electric vehicle applications.

**Fig. 12:**
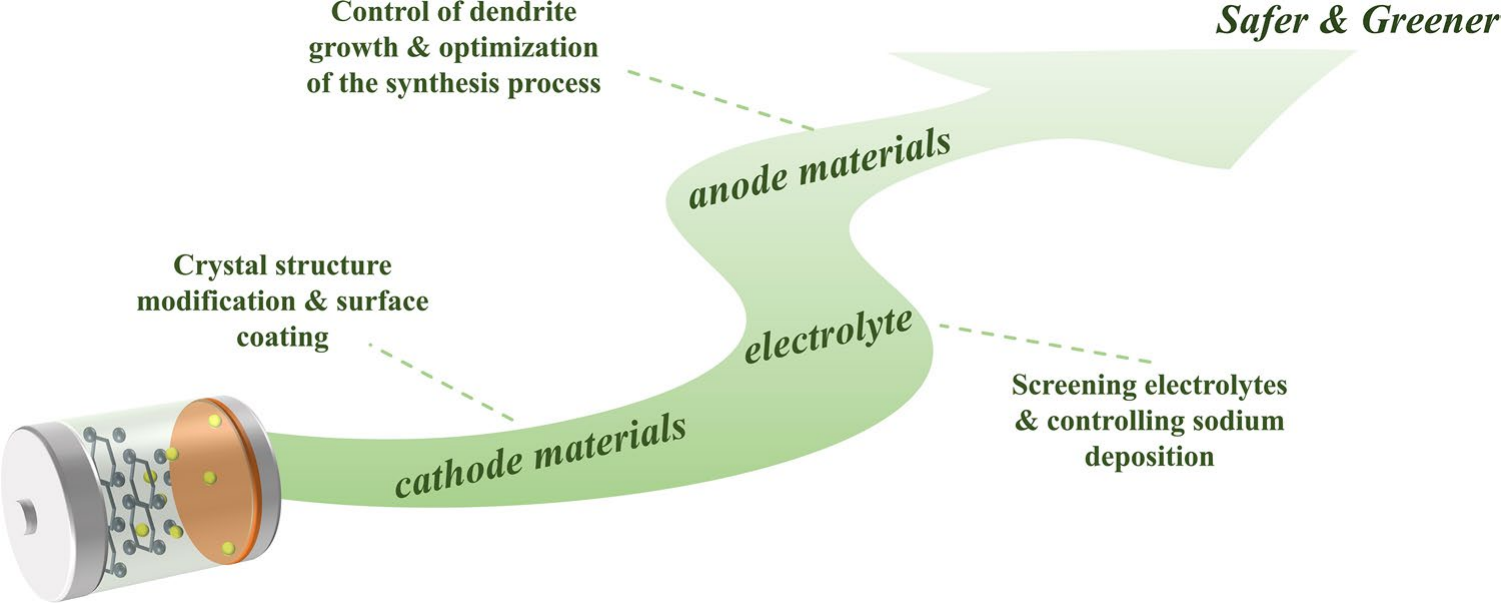
Summary of various methods of suppressing gas production inside SIBs at the present stage

**Fig. 13:**
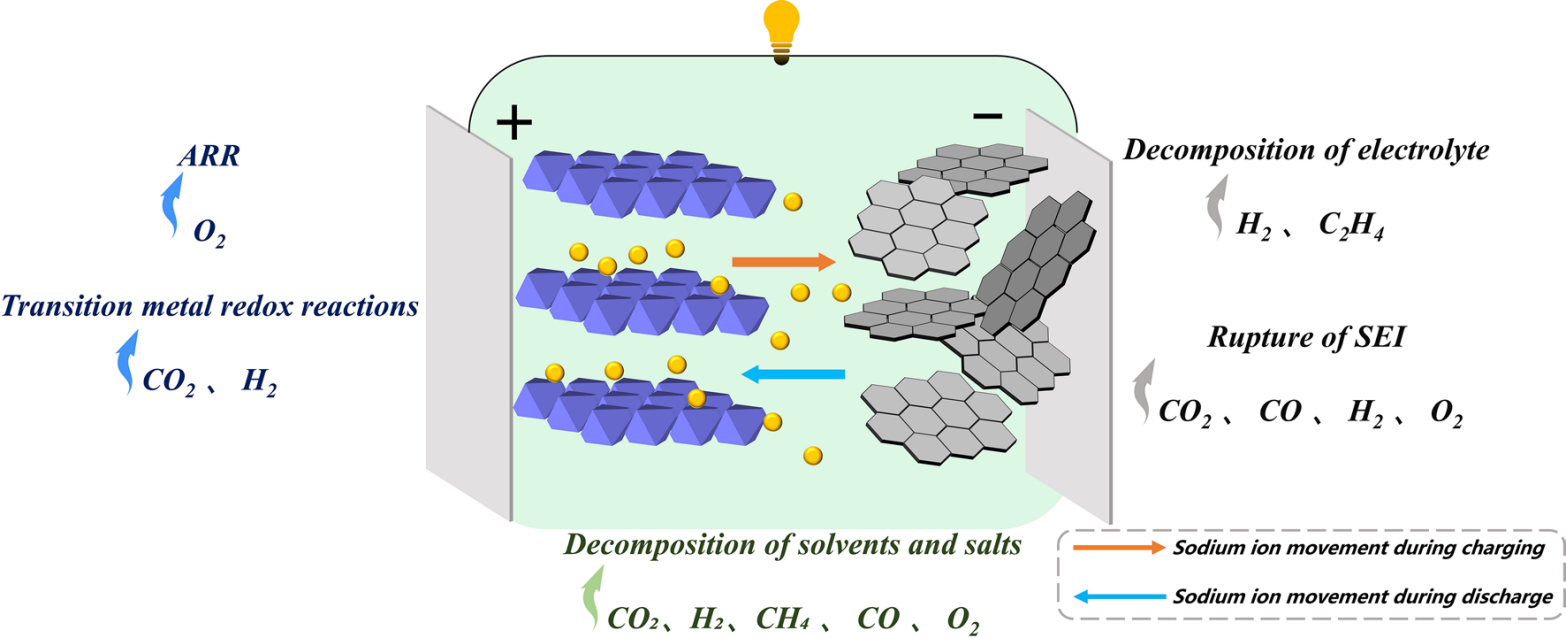
Mechanisms for the production of various gases from various reaction processes within SIBs

**Artificial intelligence-enhanced **[108]**.** Including electrolyte optimization, cathode material design, etc*.* Utilizing artificial intelligence to better and more scientifically observe and study the correlation of gas generation mechanisms within the battery. The scientifically designed gas testing processes are also a focal point of future research, aimed at elucidating the internal mechanisms of complex chemical reactions. This involves studying gas generation across the entire battery through independent investigations of the cathode and anode, thereby analyzing crossover reactions. Comprehensive discussions on gas testing correlations should systematically study normal operating conditions and thermal runaway scenarios to establish correlations between the two gas generation mechanisms. By analyzing internal correlations, more effective measures to suppress gas generation can be proposed to address gas production and battery safety issues Figs. 12 and 13.

**Enhance communication between academia and industry.** At present, the industry may have encountered technical bottlenecks in the field of SIBs with serious gas production, which can be analyzed by analyzing the data from enterprises and then guiding the direction of academic research and so on. In order to promote the development of SIBs technology and solve the major technical challenges that industry may encounter in this field, it is particularly important to strengthen communication and cooperation between academia and industry. Currently, the industry faces a series of technical bottlenecks in the R&D and production of SIBs, especially the problem of gas generation during charging and discharging, which not only affects the performance and lifetime of the batteries, but also poses a safety hazard. In order to overcome this problem, academia and industry can do data sharing and analysis, cooperative research projects, joint training of talents, and other methods. In addition, it should be noted that regular seminars and forums should be conducted and intellectual property rights should be well protected.

## References

[1] P.M. Csernica, S.S. Kalirai, W.E. Gent, K. Lim, Y.-S. Yu et al., Persistent and partially mobile oxygen vacancies in Li-rich layered oxides. Nat. Energy **6**, 642–652 (2021). 10.1038/s41560-021-00832-7

[2] F. Lin, I.M. Markus, D. Nordlund, T.-C. Weng, M.D. Asta et al., Surface reconstruction and chemical evolution of stoichiometric layered cathode materials for lithium-ion batteries. Nat. Commun. **5**, 3529 (2014). 10.1038/ncomms4529 24670975 10.1038/ncomms4529

[3] W. Song, X. Ji, Y. Zhu, H. Zhu, F. Li et al., Aqueous sodium-ion battery using a Na_3_V_2_(PO_4_)_3_ electrode. ChemElectroChem **1**, 871–876 (2014). 10.1002/celc.201300248

[4] K. Xu, Nonaqueous liquid electrolytes for lithium-based rechargeable batteries. Chem. Rev. **104**, 4303–4417 (2004). 10.1021/cr030203g 15669157 10.1021/cr030203g

[5] E. Hu, X. Yu, R. Lin, X. Bi, J. Lu et al., Evolution of redox couples in Li- and Mn-rich cathode materials and mitigation of voltage fade by reducing oxygen release. Nat. Energy **3**, 690–698 (2018). 10.1038/s41560-018-0207-z

[6] K. Luo, M.R. Roberts, N. Guerrini, N. Tapia-Ruiz, R. Hao, F. Massel, D.M. Pickup, S. Ramos, Y.-S. Liu, J. Guo, A.V. Chadwick, L.C. Duda, P.G. Bruce, Anion redox chemistry in the cobalt free 3d transition metal oxide intercalation electrode Li[Li_0.2_ Ni_0.2_ Mn_0.6_]O_2_. J. Am. Chem. Soc. **138**(35), 11211–11218 (2016). 10.1021/jacs.6b05111 27498756 10.1021/jacs.6b05111

[7] K. Luo, M.R. Roberts, R. Hao, N. Guerrini, D.M. Pickup et al., Charge-compensation in 3d-transition-metal-oxide intercalation cathodes through the generation of localized electron holes on oxygen. Nat. Chem. **8**, 684–691 (2016). 10.1038/nchem.2471 27325095 10.1038/nchem.2471

[8] X. Li, Y. Qiao, S. Guo, Z. Xu, H. Zhu et al., Direct visualization of the reversible O^2-^/O^-^ redox process in Li-rich cathode materials. Adv. Mater. **30**, e1705197 (2018). 10.1002/adma.201705197 29457283 10.1002/adma.201705197

[9] Y. Li, X. Liu, L. Wang, X. Feng, D. Ren et al., Thermal runaway mechanism of lithium-ion battery with LiNi_0.8_Mn_0.1_Co_0.1_O_2_ cathode materials. Nano Energy **85**, 105878 (2021). 10.1016/j.nanoen.2021.105878

[10] K. Chayambuka, G. Mulder, D.L. Danilov, P.H.L. Notten, From Li-ion batteries toward Na-ion chemistries: challenges and opportunities. Adv. Energy Mater. **10**, 2001310 (2020). 10.1002/aenm.202001310

[11] L. Chen, M. Fiore, J.E. Wang, R. Ruffo, D.-K. Kim et al., Readiness level of sodium-ion battery technology: a materials review. Adv. Sustain. Syst. **2**, 1700153 (2018). 10.1002/adsu.201700153

[12] C. Zhao, Q. Wang, Z. Yao, J. Wang, B. Sánchez-Lengeling et al., Rational design of layered oxide materials for sodium-ion batteries. Science **370**, 708–711 (2020). 10.1126/science.aay9972 33154140 10.1126/science.aay9972

[13] J. Tarascon, Na-ion versus Li-ion batteries: complementarity rather than competitiveness. Joule **4**, 1616–1620 (2022). 10.1016/j.joule.2020.06.003

[14] J.-Y. Hwang, S.-T. Myung, Y.-K. Sun, Sodium-ion batteries: present and future. Chem. Soc. Rev. **46**, 3529–3614 (2017). 10.1039/c6cs00776g 28349134 10.1039/c6cs00776g

[15] X. Xia, C.-F. Du, S. Zhong, Y. Jiang, H. Yu et al., Homogeneous Na deposition enabling high-energy Na-metal batteries. Adv. Funct. Mater. **32**, 2110280 (2022). 10.1002/adfm.202110280

[16] B. Yang, Z. Yang, X. Zhang, Y. Zhao, Y. Wang et al., High modulus Na_2_SiO_3_-rich solid electrolyte interphase enable long-cycle and energy-dense sodium metal battery. Adv. Funct. Mater. **34**, 2407783 (2024). 10.1002/adfm.202407783

[17] L. Li, Y. Zheng, S. Zhang, J. Yang, Z. Shao et al., Recent progress on sodium ion batteries: potential high-performance anodes. Energy Environ. Sci. **11**, 2310–2340 (2018). 10.1039/c8ee01023d

[18] H. Pan, Y.-S. Hu, L. Chen, Room-temperature stationary sodium-ion batteries for large-scale electric energy storage. Energy Environ. Sci. **6**, 2338 (2013). 10.1039/c3ee40847g

[19] K. Kawai, X.-M. Shi, N. Takenaka, J. Jang, B.M. de Boisse et al., Kinetic square scheme in oxygen-redox battery electrodes. Energy Environ. Sci. **15**, 2591–2600 (2022). 10.1039/d1ee03503g

[20] C. Zuo, D. Dong, H. Wang, Y. Sun, Y.-C. Lu, Bromide-based nonflammable electrolyte for safe and long-life sodium metal batteries. Energy Environ. Sci. **17**, 791–799 (2024). 10.1039/d3ee03332e

[21] M. Jia, Y. Qiao, X. Li, K. Jiang, H. Zhou, Unraveling the anionic oxygen loss and related structural evolution within O_3_^-^ type Na layered oxide cathodes. J. Mater. Chem. A **7**, 20405–20413 (2019). 10.1039/C9TA06186J

[22] H. Kumar, E. Detsi, D.P. Abraham, V.B. Shenoy, Fundamental mechanisms of solvent decomposition involved in solid-electrolyte interphase formation in sodium ion batteries. Chem. Mater. **28**, 8930–8941 (2016). 10.1021/acs.chemmater.6b03403

[23] J. Wang, L. Li, S. Zuo, Y. Zhang, L. Lv et al., Synchronous crystal growth and etching optimization of Prussian blue from a single iron-source as high-rate cathode for sodium-ion batteries. Electrochim. Acta **341**, 136057 (2020). 10.1016/j.electacta.2020.136057

[24] R.A. House, U. Maitra, M.A. Pérez-Osorio, J.G. Lozano, L. Jin et al., Superstructure control of first-cycle voltage hysteresis in oxygen-redox cathodes. Nature **577**, 502–508 (2020). 10.1038/s41586-019-1854-3 31816625 10.1038/s41586-019-1854-3

[25] L. Sun, Z. Wu, M. Hou, Y. Ni, H. Sun et al., Unraveling and suppressing the voltage decay of high-capacity cathode materials for sodium-ion batteries. Energy Environ. Sci. **17**, 210–218 (2024). 10.1039/d3ee02817h

[26] G. Assat, J.-M. Tarascon, Fundamental understanding and practical challenges of anionic redox activity in Li-ion batteries. Nat. Energy **3**, 373–386 (2018). 10.1038/s41560-018-0097-0

[27] X. Wang, Q. Zhang, C. Zhao, H. Li, B. Zhang et al., Achieving a high-performance sodium-ion pouch cell by regulating intergrowth structures in a layered oxide cathode with anionic redox. Nat. Energy **9**, 184–196 (2024). 10.1038/s41560-023-01425-2

[28] Y. Fang, L. Xiao, Z. Chen, X. Ai, Y. Cao et al., Recent advances in sodium-ion battery materials. Electrochem. Energ. Rev. **1**, 294–323 (2018). 10.1007/s41918-018-0008-x

[29] J. Peters, D. Buchholz, S. Passerini, M. Weil, Life cycle assessment of sodium-ion batteries. Energy Environ. Sci. **9**, 1744–1751 (2016). 10.1039/c6ee00640j

[30] E. de la Llave, E. Talaie, E. Levi, P.K. Nayak, M. Dixit et al., Improving energy density and structural stability of manganese oxide cathodes for Na-ion batteries by structural lithium substitution. Chem. Mater. **28**, 9064–9076 (2016). 10.1021/acs.chemmater.6b04078

[31] Z. Xu, J. Chen, M. Wu, C. Chen, Y. Song et al., Effects of different atmosphere on electrochemical performance of hard carbon electrode in sodium ion battery. Electron. Mater. Lett. **15**, 428–436 (2019). 10.1007/s13391-019-00143-w

[32] B. Sayahpour, W. Li, S. Bai, B. Lu, B. Han et al., Quantitative analysis of sodium metal deposition and interphase in Na metal batteries. Energy Environ. Sci. **17**, 1216–1228 (2024). 10.1039/D3EE03141A

[33] C. Gong, S.D. Pu, S. Zhang, Y. Yuan, Z. Ning et al., The role of an elastic interphase in suppressing gas evolution and promoting uniform electroplating in sodium metal anodes. Energy Environ. Sci. **16**, 535–545 (2023). 10.1039/d2ee02606f

[34] Y.-J. Guo, P.-F. Wang, Y.-B. Niu, X.-D. Zhang, Q. Li et al., Boron-doped sodium layered oxide for reversible oxygen redox reaction in Na-ion battery cathodes. Nat. Commun. **12**, 5267 (2021). 10.1038/s41467-021-25610-7 34489437 10.1038/s41467-021-25610-7 PMC8421359

[35] V. Kiran Kumar, S. Ghosh, S. Biswas, S.K. Martha, Practical realization of O3-type NaNi_0.5_Mn_0.3_Co_0.2_O_2_ cathodes for sodium-ion batteries. J. Electrochem. Soc. **167**(8), 080531 (2020). 10.1149/1945-7111/ab8ed5

[36] C. Li, F. Geng, B. Hu, B. Hu, Anionic redox in Na-based layered oxide cathodes: a review with focus on mechanism studies. Mater. Today Energy **17**, 100474 (2020). 10.1016/j.mtener.2020.100474

[37] Y. Wang, J. Jin, X. Zhao, Q. Shen, X. Qu et al., Unexpected elevated working voltage by Na^+^/vacancy ordering and stabilized sodium-ion storage by transition-metal honeycomb ordering. Angew. Chem. Int. Ed. **63**, e202409152 (2024). 10.1002/anie.202409152 10.1002/anie.202409152 38923635

[38] Y. Zhao, Q. Liu, X. Zhao, D. Mu, G. Tan et al., Structure evolution of layered transition metal oxide cathode materials for Na-ion batteries: Issues, mechanism and strategies. Mater. Today **62**, 271–295 (2023). 10.1016/j.mattod.2022.11.024

[39] S. Zhang, X. Li, Y. Su, Y. Yang, H. Yu et al., Four-In-one strategy to boost the performance of Na_x_[Ni, Mn]O_2_. Adv. Funct. Mater. **33**, 2301568 (2023). 10.1002/adfm.202301568

[40] J. Do, I. Kim, H. Kim, Y. Jung, Towards stable Na-rich layered transition metal oxides for high energy density sodium-ion batteries. Energy Storage Mater. **25**, 62–69 (2020). 10.1016/j.ensm.2019.10.031

[41] Y. Wang, X. Wang, X. Li, Yu. Ruizhi, M. Chen, K. Tang, X. Zhang, The novel P3-type layered Na_0.65_Mn_0.75_Ni_0.25_O_2_ oxides doped by non-metallic elements for high performance sodium-ion batteries. Chem. Eng. J. **360**, 139–147 (2019). 10.1016/j.cej.2018.11.214

[42] Z. Wu, Y. Ni, S. Tan, E. Hu, L. He et al., Realizing high capacity and zero strain in layered oxide cathodes via lithium dual-site substitution for sodium-ion batteries. J. Am. Chem. Soc. **145**, 9596–9606 (2023). 10.1021/jacs.3c00117 37058227 10.1021/jacs.3c00117

[43] R. Mogensen, D. Brandell, R. Younesi, Solubility of the solid electrolyte interphase (SEI) in sodium ion batteries. ACS Energy Lett. **1**, 1173–1178 (2016). 10.1021/acsenergylett.6b00491

[44] B. Lee, E. Paek, D. Mitlin, S.W. Lee, Sodium metal anodes: emerging solutions to dendrite growth. Chem. Rev. **119**, 5416–5460 (2019). 10.1021/acs.chemrev.8b00642 30946573 10.1021/acs.chemrev.8b00642

[45] T. Cai, M. Cai, J. Mu, S. Zhao, H. Bi et al., High-entropy layered oxide cathode enabling high-rate for solid-state sodium-ion batteries. Nano-Micro Lett. **16**, 10 (2023). 10.1007/s40820-023-01232-0 10.1007/s40820-023-01232-0 PMC10635981 37943381

[46] X. Chen, X. Shen, B. Li, H.-J. Peng, X.-B. Cheng et al., Ion–solvent complexes promote gas evolution from electrolytes on a sodium metal anode. Angew. Chem. Int. Ed. **57**, 734–737 (2018). 10.1002/anie.201711552 10.1002/anie.201711552 29178154

[47] X. Zheng, L. Huang, X. Ye, J. Zhang, F. Min et al., Critical effects of electrolyte recipes for Li and Na metal batteries. Chem **7**, 2312–2346 (2021). 10.1016/j.chempr.2021.02.025

[48] S. Lin, H. Zhang, C. Shu, W. Hua, X. Wang et al., Research progress and perspectives on pre-sodiation strategies for sodium-ion batteries. Adv. Funct. Mater. **34**, 2409628 (2024). 10.1002/adfm.202409628

[49] F. Wang, N. Zhang, X. Zhao, L. Wang, J. Zhang et al., Realizing a high-performance Na-storage cathode by tailoring ultrasmall Na_2_FePO_4_F nanoparticles with facilitated reaction kinetics. Adv. Sci. **6**, 1900649 (2019). 10.1002/advs.201900649 10.1002/advs.201900649 PMC6662290 31380194

[50] Z. Lu, H. Yang, Y. Guo, P. He, S. Wu et al., Electrolyte sieving chemistry in suppressing gas evolution of sodium-metal batteries. Angew. Chem. Int. Ed. **61**, e202206340 (2022). 10.1002/anie.202206340 10.1002/anie.202206340 35607934

[51] W. Liu, X. Chen, C. Zhang, H. Xu, X. Sun et al., Gassing in Sn-anode sodium-ion batteries and its remedy by metallurgically prealloying Na. ACS Appl. Mater. Interfaces **11**, 23207–23212 (2019). 10.1021/acsami.9b05005 31140773 10.1021/acsami.9b05005

[52] H.-R. Yao, X.-G. Yuan, X.-D. Zhang, Y.-J. Guo, L. Zheng et al., Excellent air storage stability of Na-based transition metal oxide cathodes benefiting from enhanced Na−O binding energy. Energy Storage Mater. **54**, 661–667 (2023). 10.1016/j.ensm.2022.11.005

[53] X. Wu, Z. Piao, M. Zhang, G. Lu, C. Li et al., *In situ* construction of a multifunctional interphase enabling continuous capture of unstable lattice oxygen under ultrahigh voltages. J. Am. Chem. Soc. **146**, 14036–14047 (2024). 10.1021/jacs.4c02345 38725301 10.1021/jacs.4c02345

[54] G. Sahu, Z. Lin, J. Li, Z. Liu, N. Dudney et al., Air-stable, high-conduction solid electrolytes of arsenic-substituted Li_4_SnS_4_. Energy Environ. Sci. **7**, 1053–1058 (2014). 10.1039/c3ee43357a

[55] H. Wang, Y. Chen, Z.D. Hood, G. Sahu, A.S. Pandian et al., An air-stable Na_3_SbS_4_ superionic conductor prepared by a rapid and economic synthetic procedure. Angew. Chem. Int. Ed. **55**, 8551–8555 (2016). 10.1002/anie.201601546 10.1002/anie.201601546 27246874

[56] Z. Liu, Z. Lu, S. Guo, Q.-H. Yang, H. Zhou, Toward high performance anodes for sodium-ion batteries: from hard carbons to anode-free systems. ACS Cent. Sci. **9**, 1076–1087 (2023). 10.1021/acscentsci.3c00301 37396865 10.1021/acscentsci.3c00301 PMC10311662

[57] Z. Song, G. Zhang, X. Deng, Y. Tian, X. Xiao et al., Strongly coupled interfacial engineering inspired by robotic arms enable high-performance sodium-ion capacitors. Adv. Funct. Mater. **32**, 2205453 (2022). 10.1002/adfm.202205453

[58] X. Xiao, X. Duan, Z. Song, X. Deng, W. Deng et al., High-throughput production of cheap mineral-based heterostructures for high power sodium ion capacitors. Adv. Funct. Mater. **32**, 2110476 (2022). 10.1002/adfm.202110476

[59] Z. Song, G. Zhang, X. Deng, K. Zou, X. Xiao et al., Ultra-low-dose pre-metallation strategy served for commercial metal-ion capacitors. Nano-Micro Lett. **14**, 53 (2022). 10.1007/s40820-022-00792-x 10.1007/s40820-022-00792-x PMC8800971 35092494

[60] X.-L. Li, T. Wang, Y. Yuan, X.-Y. Yue, Q.-C. Wang et al., Whole-voltage-range oxygen redox in P2-layered cathode materials for sodium-ion batteries. Adv. Mater. **33**, e2008194 (2021). 10.1002/adma.202008194 33645858 10.1002/adma.202008194

[61] L. Mu, R. Lin, R. Xu, L. Han, S. Xia et al., Oxygen release induced chemomechanical breakdown of layered cathode materials. Nano Lett. **18**, 3241–3249 (2018). 10.1021/acs.nanolett.8b01036 29667835 10.1021/acs.nanolett.8b01036

[62] J. Xu, M. Sun, R. Qiao, S.E. Renfrew, L. Ma et al., Elucidating anionic oxygen activity in lithium-rich layered oxides. Nat. Commun. **9**, 947 (2018). 10.1038/s41467-018-03403-9 29507369 10.1038/s41467-018-03403-9 PMC5838240

[63] T. Cui, L. Liu, Y. Xiang, C. Sheng, X. Li, F. Yongzhu, Facilitating an ultrastable O3-type cathode for 4.5 V sodium-ion batteries *via* a dual-reductive coupling mechanism. J. Am. Chem. Soc. **146**, 13924–13933 (2024). 10.1021/jacs.4c01787 38723613 10.1021/jacs.4c01787

[64] Y. Zhang, M. Wu, J. Ma, G. Wei, Y. Ling et al., Revisiting the Na_2/3_Ni_1/3_Mn_2/3_O_2_ cathode: oxygen redox chemistry and oxygen release suppression. ACS Cent. Sci. **6**, 232–240 (2020). 10.1021/acscentsci.9b01166 32123741 10.1021/acscentsci.9b01166 PMC7047265

[65] Y. Wang, X. Zhao, J. Jin, Q. Shen, Y. Hu et al., Boosting the reversibility and kinetics of anionic redox chemistry in sodium-ion oxide cathodes *via* reductive coupling mechanism. J. Am. Chem. Soc. **145**, 22708–22719 (2023). 10.1021/jacs.3c08070 37813829 10.1021/jacs.3c08070

[66] J. Peng, W. Zhang, Q. Liu, J. Wang, S. Chou et al., Prussian blue analogues for sodium-ion batteries: past, present, and future. Adv. Mater. **34**, e2108384 (2022). 10.1002/adma.202108384 34918850 10.1002/adma.202108384

[67] J.F. Peters, M. Weil, Aqueous hybrid ion batteries–An environmentally friendly alternative for stationary energy storage? J. Power Sources **364**, 258–265 (2017). 10.1016/j.jpowsour.2017.08.041

[68] J. Qian, C. Wu, Y. Cao, Z. Ma, Y. Huang et al., Prussian blue cathode materials for sodium-ion batteries and other ion batteries. Adv. Energy Mater. **8**, 1702619 (2018). 10.1002/aenm.201702619

[69] F. Feng, S. Chen, X.-Z. Liao, Z.-F. Ma, Hierarchical hollow Prussian blue rods synthesized via self-sacrifice template as cathode for high performance sodium ion battery. Small Meth. **3**, 1800259 (2019). 10.1002/smtd.201800259

[70] Y. You, X.-L. Wu, Y.-X. Yin, Y.-G. Guo, High-quality Prussian blue crystals as superior cathode materials for room-temperature sodium-ion batteries. Energy Environ. Sci. **7**, 1643–1647 (2014). 10.1039/C3EE44004D

[71] S.L. Dreyer, F.M. Maddar, A. Kondrakov, J. Janek, I. Hasa et al., Elucidating gas evolution of Prussian white cathodes for sodium-ion battery application: the effect of electrolyte and moisture. Batter. Supercaps **7**, e202300595 (2024). 10.1002/batt.202300595

[72] L. Zhang, C. Tsolakidou, S. Mariyappan, J.-M. Tarascon, S. Trabesinger, Unraveling gas evolution in sodium batteries by online electrochemical mass spectrometry. Energy Storage Mater. **42**, 12–21 (2021). 10.1016/j.ensm.2021.07.005

[73] H. Zhang, X. Tan, H. Li, S. Passerini, W. Huang, Assessment and progress of polyanionic cathodes in aqueous sodium batteries. Energy Environ. Sci. **14**, 5788–5800 (2021). 10.1039/d1ee01392k

[74] S. Chen, C. Wu, L. Shen, C. Zhu, Y. Huang et al., Challenges and perspectives for NASICON-type electrode materials for advanced sodium-ion batteries. Adv. Mater. **29**, 1700431 (2017). 10.1002/adma.201700431 10.1002/adma.201700431 28626908

[75] H. Li, T. Wang, X. Wang, G. Li, J. Shen et al., MOF-derived Al-doped Na_2_FePO_4_F/mesoporous carbon nanonetwork composites as high-performance cathode material for sodium-ion batteries. Electrochim. Acta **373**, 137905 (2021). 10.1016/j.electacta.2021.137905

[76] Z. Xiong, X. Nie, B. Zhang, Z. Wei, Na_2_S cathodes enabling safety room temperature sodium sulfur batteries. Batter. Supercaps **7**, e202300503 (2024). 10.1002/batt.202300503

[77] H.A. Adeoye, S. Tennison, J.F. Watts, C. Lekakou, An investigation into electrolytes and cathodes for room-temperature sodium–sulfur batteries. Batteries **10**, 216 (2024). 10.3390/batteries10060216

[78] S.I. Kim, W.I. Park, K. Jung, C.-S. Kim, An innovative electronically-conducting matrix of the cathode for sodium sulfur battery. J. Power Sources **320**, 37–42 (2016). 10.1016/j.jpowsour.2016.04.041

[79] S. Lu, Y. Liu, J. Xu, S. Weng, J. Xue et al., Design towards recyclable micron-sized Na_2_S cathode with self-refinement mechanism. Nat. Commun. **15**, 9995 (2024). 10.1038/s41467-024-54316-9 39557850 10.1038/s41467-024-54316-9 PMC11573996

[80] D.P. DiVincenzo, E.J. Mele, Cohesion and structure in stage-1 graphite intercalation compounds. Phys. Rev. B Condens. Matter **32**, 2538–2553 (1985). 10.1103/physrevb.32.2538 9937330 10.1103/physrevb.32.2538

[81] R. Alcántara, J. Jiménez-Mateos, P. Lavela, J. Tirado, Carbon black: a promising electrode material for sodium-ion batteries. Electrochem. Commun. **3**, 639–642 (2014). 10.1016/S1388-2481(01)00244-2

[82] P. Thomas, J. Ghanbaja, D. Billaud, Electrochemical insertion of sodium in pitch-based carbon fibres in comparison with graphite in NaClO_4_–ethylene carbonate electrolyte. Electrochim. Acta **45**, 423–430 (1999). 10.1016/S0013-4686(99)00276-5

[83] D.A. Stevens, J.R. Dahn, An *in situ* small-angle X-ray scattering study of sodium insertion into a nanoporous carbon anode material within an operating electrochemical cell. J. Electrochem. Soc. **147**, 4428 (2000). 10.1149/1.1394081

[84] Y. Wang, M. Li, Y. Zhang, N. Zhang, Hard carbon for sodium storage: mechanism and performance optimization. Nano Res. **17**, 6038–6057 (2024). 10.1007/s12274-024-6546-0

[85] N. Yabuuchi, M. Kajiyama, J. Iwatate, H. Nishikawa, S. Hitomi et al., P2-type Na_*x*_[Fe_1/2_Mn_1/2_]O_2_ made from earth-abundant elements for rechargeable Na batteries. Nat. Mater. **11**, 512–517 (2012). 10.1038/nmat3309 22543301 10.1038/nmat3309

[86] Q. Liu, D. Mu, B. Wu, L. Wang, L. Gai et al., Density functional theory research into the reduction mechanism for the solvent/additive in a sodium-ion battery. ChemSusChem **10**, 786–796 (2017). 10.1002/cssc.201601356 27897399 10.1002/cssc.201601356

[87] X. Deng, Y. Huang, Y. Han, J. Du, J. Tian et al., Abnormal gas generation during first discharge process of sodium ion battery. Angew. Chem. Int. Ed. **63**, e202412222 (2024). 10.1002/anie.202412222 10.1002/anie.202412222 39106271

[88] J. Luo, M. Yang, D. Wang, J. Zhang, K. Song et al., A fast Na-ion conduction polymer electrolyte *via* triangular synergy strategy for quasi-solid-state batteries. Angew. Chem. Int. Ed. **62**, e202315076 (2023). 10.1002/anie.202315076 10.1002/anie.202315076 37960950

[89] J. Ge, C. Ma, Y. Zhang, P. Ma, J. Zhang et al., Edge electron effect induced high-entropy SEI for durable anode-free sodium batteries. Adv. Mater. (2024). 10.1002/adma.202413253 10.1002/adma.202413253 39568239

[90] J. Xue, H. Zhang, J. Chen, K. Fang, Y. Chen et al., Unlocking the Na-storage behavior in hard carbon anode by mass spectrometry. Nano Lett. **24**, 9839–9845 (2024). 10.1021/acs.nanolett.4c01620 39087826 10.1021/acs.nanolett.4c01620

[91] W. Zhang, F. Zhang, F. Ming, H.N. Alshareef, Sodium-ion battery anodes: status and future trends. EnergyChem **1**, 100012 (2019). 10.1016/j.enchem.2019.100012

[92] S. Lin, Z. Yang, J. Chen, Y. Qiao, L. Li et al., Functional electrolyte additives for sodium-ion and sodium-metal batteries: progress and perspectives. Adv. Funct. Mater. **34**, 2400731 (2024). 10.1002/adfm.202400731

[93] Y. Lee, J. Lee, J. Lee, K. Kim, A. Cha et al., Fluoroethylene carbonate-based electrolyte with 1 M sodium bis(fluorosulfonyl)imide enables high-performance sodium metal electrodes. ACS Appl. Mater. Interfaces **10**, 15270–15280 (2018). 10.1021/acsami.8b02446 29648435 10.1021/acsami.8b02446

[94] G. Deysher, J.A.S. Oh, Y.-T. Chen, B. Sayahpour, S.-Y. Ham et al., Design principles for enabling an anode-free sodium all-solid-state battery. Nat. Energy **9**, 1161–1172 (2024). 10.1038/s41560-024-01569-9

[95] D. Aurbach, A short review of failure mechanisms of lithium metal and lithiated graphite anodes in liquid electrolyte solutions. Solid State Ion. **148**, 405–416 (2002). 10.1016/s0167-2738(02)00080-2

[96] C. Zhao, L. Liu, X. Qi, Y. Lu, F. Wu et al., Solid-state sodium batteries. Adv. Energy Mater. **8**, 1703012 (2018). 10.1002/aenm.201703012

[97] N. Yabuuchi, R. Hara, M. Kajiyama, K. Kubota, T. Ishigaki et al., New O2/P2-type Li-excess layered manganese oxides as promising multi-functional electrode materials for rechargeable Li/Na batteries. Adv. Energy Mater. **4**, 1301453 (2014). 10.1002/aenm.201301453

[98] K. Du, J. Zhu, G. Hu, H. Gao, Y. Li et al., Exploring reversible oxidation of oxygen in a manganese oxide. Energy Environ. Sci. **9**, 2575–2577 (2016). 10.1039/c6ee01367h

[99] L. Yu, X. He, B. Peng, F. Wang, N. Ahmad et al., Nonmetal substitution in interstitial site of O3- NaNi_0.5_Mn_0.5_O_2_ induces the generation of a nearly zero strain P2&O3 biphasic structure as ultrastable sodium-ion cathode. Adv. Funct. Mater. **34**, 2406771 (2024). 10.1002/adfm.202406771

[100] Y. Zhu, Z. Zhang, J. Bao, S. Zeng, W. Nie et al., Multi-metal doped high capacity and stable Prussian blue analogue for sodium ion batteries. Int. J. Energy Res. **44**, 9205–9212 (2020). 10.1002/er.5576

[101] M. Gu, I. Belharouak, J. Zheng, H. Wu, J. Xiao et al., Formation of the spinel phase in the layered composite cathode used in Li-ion batteries. ACS Nano **7**, 760–767 (2013). 10.1021/nn305065u 23237664 10.1021/nn305065u

[102] Y. Liu, X. Fang, A. Zhang, C. Shen, Q. Liu et al., Layered P2-Na_2/3_[Ni_1/3_Mn_2/3_]O_2_ as high-voltage cathode for sodium-ion batteries: The capacity decay mechanism and Al_2_O_3_ surface modification. Nano Energy **27**, 27–34 (2016). 10.1016/j.nanoen.2016.06.026

[103] R. Rodriguez, K.E. Loeffler, S.S. Nathan, J.K. Sheavly, A. Dolocan et al., In situ optical imaging of sodium electrodeposition: effects of fluoroethylene carbonate. ACS Energy Lett. **2**, 2051–2057 (2017). 10.1021/acsenergylett.7b00500

[104] M. Xia, H. Chen, Z. Zheng, Q. Meng, A. Zhao et al., Sodium-difluoro(oxalato)borate-based electrolytes for long-term cycle life and enhanced low-temperature sodium-ion batteries. Adv. Energy Mater. (2024). 10.1002/aenm.202403306

[105] M. Li, J. Jiang, Y. Chen, S. Huang, X. Liu et al., A novel anion receptor additive for-40 °C sodium metal batteries by anion/cation solvation engineering. Angew. Chem. Int. Ed. **64**, e202413806 (2025). 10.1002/anie.202413806 10.1002/anie.202413806 39417785

[106] Q. Lu, A. Omar, L. Ding, S. Oswald, M. Hantusch et al., A facile method to stabilize sodium metal anodes towards high-performance sodium batteries. J. Mater. Chem. A **9**, 9038–9047 (2021). 10.1039/d1ta00066g

[107] Y. Chen, X. Li, K. Park, W. Lu, C. Wang et al., Nitrogen-doped carbon for sodium-ion battery anode by self-etching and graphitization of bimetallic MOF-based composite. Chem **3**, 152–163 (2017). 10.1016/j.chempr.2017.05.021

[108] P. Xu, G. Li, Y. Zheng, J.C.H. Fung, A. Chen et al., Fertilizer management for global ammonia emission reduction. Nature **626**, 792–798 (2024). 10.1038/s41586-024-07020-z 38297125 10.1038/s41586-024-07020-z

[109] J. Yue, L. Lin, L. Jiang, Q. Zhang, Y. Tong et al., Interface concentrated-confinement suppressing cathode dissolution in water-in-salt electrolyte. Adv. Energy Mater. **10**, 2000665 (2020). 10.1002/aenm.202000665

